# An Electrochemical Study of the Corrosion Behaviour of the Polished Atomic Diffusion Additive Manufactured 17-4PH Stainless Steel Using Centrifugal Mass Finishing Method in Saltwater

**DOI:** 10.3390/ma18225148

**Published:** 2025-11-12

**Authors:** Juan Ignacio Ahuir-Torres, Xiaoxiao Liu, Jackson Chadwick, Tahsin Tecelli Öpöz

**Affiliations:** School of Engineering, Liverpool John Moores University, Liverpool L3 3AF, UK; j.w.chadwick@2024.ljmu.ac.uk (J.C.); t.t.opoz@ljmu.ac.uk (T.T.Ö.)

**Keywords:** atomic diffusion additive manufacturing, 17-4PH stainless steel, centrifugal mass polishing, surface roughness, corrosion behaviour

## Abstract

Additive manufacturing (AM) enables the fabrication of complex geometries and high-performance alloys such as 17-4PH stainless steel. However, the surface defects inherent to AM components compromise corrosion resistance. The post surface treatment can reduce and eliminate these defects. This study examines the effect of centrifugal mass finishing on the corrosion behaviour of 17-4PH stainless steel produced by AM. Corrosion behaviour of the samples in a 0.6 M NaCl solution is assessed using electrochemical technique testing, including asymmetry electrochemical nose, potentiodynamic polarisation curves, electrochemical impedance spectroscopy, and Mott-Schottky. All electrochemical testing were conducted in concordance with the specifications of ASTM standards. The electrochemical impedance spectroscopy were performed over period ranging from 2 h to 96 h with intervals of approximately one day. Finished specimens exhibit significantly improved corrosion resistance compared with as-built counterparts. Notably, the polished surfaces demonstrate spontaneous oxidised layer recovery between −0.297 V and 0 V, indicative of the enhancement of the protection during early immersion stages. This behaviour is attributed to surface modifications induced by the finishing process, including reduced roughness in 78% and imperfections. These findings highlight the importance of optimising post-processing protocols to improve the durability of AM stainless steels in aggressive environments.

## 1. Introduction

Additive manufacturing (AM), particularly metal-based 3D printing technologies, including Direct Metal Laser Sintering (DMLS), Selective Laser Melting (SLM), and Fused Filament Fabrication (FFF), has transformed the way complex, lightweight structures are produced for many sectors across industries ranging from aerospace to biomedical engineering [1,2]. Unlike conventional subtractive processes, AM employs a layer-by-layer deposition strategy that enables the fabrication of intricate geometries with reduced material waste, enhanced design freedom, and compatibility with automated production [3]. Within the wide range of metal AM techniques, metal-based fused filament fabrication (MB-FFF), represented by processes such as Atomic Diffusion Additive Manufacturing (ADAM) and Bound Metal Deposition (BMD), offers a cost-effective alternative to powder-bed systems. This approach addresses challenges such as high powder costs, stringent safety requirements, and the need for post-build powder removal [4].

Despite these advantages, AM-fabricated components frequently exhibit surface defects, including high surface roughness (Ra > 10 µm), porosity, voids, and unmelted particles. Moreover, the rapid solidification and layer-by-layer deposition characteristic of AM processes also introduce microstructural anisotropy, stair-stepping effects, and residual stresses, all of which can compromise the integrity of protective passive oxide layers [5]. These microstructural and topographical irregularities act as stress concentrators and pathways for corrosion, thereby weakening the stability of protective surface films. Consequently, such features can significantly reduce mechanical performance and corrosion resistance, particularly when components are deployed in aggressive service environments [6].

In precipitation-hardening stainless steels, such as 17-4PH, an alloy widely used for its high mechanical strength and moderate corrosion resistance, these defects are of particular concern. As they frequently serve as initiation sites for localised corrosion mechanisms such as pitting and crevice corrosion, especially in chloride-rich environments [7,8]. For example, Sander et al. [8] demonstrated that as-built AM 316L stainless steel exhibited approximately 30% lower corrosion resistance than its wrought counterpart, with surface roughness directly linked to accelerated corrosion. This evidence highlights the importance of effective post-processing to ensure that AM stainless steels meet the stringent requirements of critical applications. However, conventional finishing techniques such as machining and grinding are often impractical for components with the intricate geometries typical of AM [9].

To overcome these limitations, mass finishing (MF) techniques, including vibratory finishing, abrasive flow machining, and centrifugal disc finishing, have gained increasing attention for their ability to uniformly refine surfaces with minimal material removal [10,11]. Among these, centrifugal disc finishing is a high-energy MF method in which components are immersed in a rotating chamber filled with abrasive media; the helical motion of the media generates an intense and uniform grinding action [10]. Previous studies on mass finished alloys such as Ti-6Al-4V and Inconel 718 have demonstrated substantial reductions in surface roughness (Ra < 1 µm) and corresponding improvements in fatigue life [12,13]. However, the electrochemical effects of MF, particularly its potential to enhance corrosion resistance in AM stainless steels, remain insufficiently explored. For instance, recent work by Opoz et al. [12] showed that centrifugal disc finishing improved isotropy and ultimate tensile strength (UTS) in 17-4PH stainless steel, but highlighted the need for further investigation into surface integrity and corrosion behaviour [14]. Similarly, Kareem et al. [14] reported that 17-4PH stainless steel fabricated via the sinter-based method exhibited relatively poor corrosion resistance, primarily due to high surface roughness and cracks. These surface imperfections were shown to promote the formation of microgalvanic cells and local oxygen concentration gradients, thereby accelerating crevice corrosion. Mwema et al. [15] also investigated the corrosion behaviour of 17-4PH stainless steel fabricated through material extrusion additive manufacturing, focusing on the influence of processing parameters on corrosion resistance. Their findings indicated that certain fabrication conditions resulted in the formation of an austenitic microstructure, which reduced corrosion resistance.

Although previous studies have explored AM process parameters, microstructural features, and mechanical properties [13,14], little attention has been given to the relationship between post-processing and corrosion performance. Additive manufacturing of 17-4PH stainless steel frequently results in surface defects, porosity, and residual stresses, which can adversely affect corrosion resistance. Post-processing techniques such as heat treatment and shot peening have been shown to enhance corrosion resistance by refining microstructure and inducing compressive residual stresses; however, surface roughness introduced by some post-processing methods can negatively affect corrosion performance if not optimised [16,17]. Surface finishing approaches, including machining and polishing, can reduce surface irregularities and mitigate localised corrosion [12], while studies on laser powder bed fusion 17-4PH stainless steel highlight that optimised post-processing is crucial to balance corrosion resistance with mechanical performance by addressing inherent porosity and surface defects [18,19]. Despite these insights, the corrosion behaviour of 17-4PH fabricated using metal-based fused filament (MBFF) remains largely unaddressed, both in as-built condition and after centrifugal disc mass finishing (CDMF) process. Furthermore, the temporal evolution of corrosion behaviour of MBFFed 17-4PH has yet to be systematically explored, and the influence of MBFF processing on the corrosion performance of metallic materials has been barely investigated. The present study seeks to address these gaps by examining the effect of CDMF on the surface characteristics and electrochemical behaviour of 17-4PH stainless steel tensile specimens fabricated via MBFF. Specifically, the study aims to evaluate the influence of the finishing process on the corrosion behaviour of the MBFFed 17-4PH in 0.6 M NaCl through several electrochemical techniques, including asymmetric electrochemical noise, potentiodynamic polarisation curve, electrochemical impedance spectroscopy, and Mott-Schottky method.

## 2. Experimental Setup

### 2.1. Sample Preparation

Samples were produced from 17 to 4PH stainless steel using the Markforged Metal X system, an ADAM technique [12]. The 17-4PH SS filament supplied by Markforged consisted of 60 vol.% stainless steel powder with 40 vol.% binder composed of paraffin wax and polyethylene [12,20]. The chemical composition of the 17-4PH stainless steel is given in Table 1. The samples were designed with parallel-sided gauge sections to enable reliable surface and electrochemical evaluations. The initial geometries were modelled in SolidWorks 2025 SP4.1 and exported as stereolithography (STL) files, which were subsequently processed using Markforged’s proprietary slicing software to generate the required deposition toolpaths. A full (100%) nominal infill density was selected to ensure mechanical integrity and minimise internal porosity. During printing, the filament was extruded through a 0.4 mm twin-nozzle system to produce “green” parts, with a ceramic interface material deposited between supports for easy removal after sintering. The printed parts were then subjected to a two-stage post-processing route: solvent-based debinding in Opteon SF 79 solution (Markforged Wash-1) until 4.1% weight loss, followed by high-temperature sintering in a Markforged Sinter-1 furnace under an argon atmosphere with 3% hydrogen. The sintering process lasted approximately 27 h, with the maximum temperature estimated at around 1100 °C [12]. This stage promoted diffusion bonding between metal particles, resulting in dense, fully metallic as-built components that were subsequently used for centrifugal mass finishing and corrosion tests without further heat treatment.

### 2.2. Centrifugal Disc Finishing and Measurements

**Figure 1:**
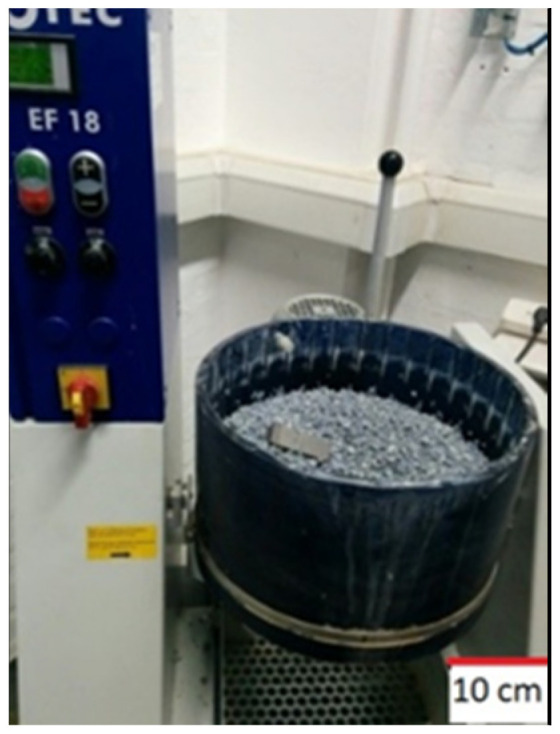
Diagrams of a centrifugal disc finishing machine.

In the post-processing phase, surface finishing was performed using an OTEC CF18 Element Series centrifugal disc finishing machine (Figure 1). The finishing bowl was loaded with 18 tape water litres of ceramic abrasive media (product provided by OTEC, Straubenhardt–Conweiler, Germany), and the samples were placed into the finishing bowl to ensure uniform contact with the media throughout the process. Each sample underwent finishing for three hours at a rotational speed of 310 rpm. After completion of the finishing treatment, the samples were cleaned in an ultrasonic bath at 30 °C for 5 min to remove any residual debris or contaminants that could interfere with subsequent analyses [21].

### 2.3. Corrosion Behaviour Evaluation

All electrochemical tests were conducted using a potentio/galvanostat (Interface1010E, Gamry Instruments Inc., Warminster, PA, USA) operated through Gamry Framework software version 7.4 and data were analysed using Gamry Echem Analyst software version 7.4, a three-electrode cell was employed for all electrochemical tests, consisting of a reference (±10^−3^ M KCl silver/silver chloride (Ag/AgCl 3 ± 10^−3^ M KCl) of double-junction (EDT direct ion Ltd., Dover, UK), counter (platinum wire of 99.999% purity with a 0.7 ± 10^−3^ mm diameter (Heimerle + Meule Group, Pforzheim, Germany) and working (samples) electrode. All corrosion tests were conducted in 0.6 ± 10^−3^ M sodium chloride (NaCl) (Sigma–Aldrich Merck, Darmstadt, Germany) solution at room temperature (23 ± 2 °C) and pH 7.

The electrochemical techniques employed included asymmetric electrochemical noise (AEN), potentiodynamic polarisation curve (PPC), electrochemical impedance spectroscopy (EIS), and Mott-Schoktty. AEN is a combination of an open-circuit potential (OCP) and a zero-resistance ammeter (ZRA). AEN tests were carried out for 3600 ± 10^−3^ s with 0.05 s acquisition interval (STP1277-EB ASTM [22]). PPC tests were conducted using an initial potential of −0.3 ± 10^−3^ V vs. open circuit potential after 1 ± 2.7 10^−7^ h of immersion in 0.6 ± 10^−3^ M NaCl, with a voltage scan rate of 0.167 ± 10^−6^ mVs^−1^, a current density limit of 5 ± 10^−12^ mAcm^−2^, and a return potential of 3 ± 10^−6^ V vs. the reference electrode (ASTM G61 [23]). EIS measurements were performed with 10 ± 10^−3^ mV root mean square potential amplitude, over a frequency range from 10^−2^ ± 10^−3^ Hz to 10^5^ ± 10^−3^ Hz with 10 points per frequency decade. EIS tests were conducted at 2 ± 2.7 10^−7^, 24 ± 2.7 10^−7^, 48 ± 2.7 10^−7^, 72 ± 2.7 10^−7^, and 96 ± 2.7 10^−7^ h of solution immersion (ASTM G106 [24]). The Mott-Schoktty method was carried out using OCP after 15 ± 8.1 10^−6^ min of immersion as the initial potential, over a range from −1 ± 10^−6^ V to 1 ± 10^−6^ V relative to OCP, with 10 ± 10^−3^ mV root mean square potential amplitude, a scan rate of 50 ± 10^−3^ mVs^−1^, and a frequency of 10^3^ ± 10^−3^ Hz (ASTM [25]).

Notably, all measurements were repeated at least three times to ensure reproducibility and statistical significance.

### 2.4. Material Characterisation

The surfaces and microstructures of the samples, both before and after corrosion testing (PPC), were examined using SEM (Hitachi TM4000plus, Krefeld, Germany). SEM was operated at an accelerating voltage of 15 ± 10^−3^ kV, a beam current of 70 ± 10^−6^ μA, and a 2 ± 10^−2^ μm beam spot. Imaging was conducted with both secondary and backscattered electrons. The microstructure of the samples was revealed through sequential mechanical polishing followed by chemical etching. The mechanical polishing was carried out using silicon carbide (SiC) abrasive papers of 600 and 1200 grits, followed by polishing with 3 μm and 1 μm diamond paste. Final mirror finishing was achieved with colloidal silica suspension (50% in volume in distilled water) containing particles of 40 nm. The chemical etching was performed by immersing the polished samples for 30 ± 10^−3^ s in an etchant solution consisting of 40 ± 10^−3^ mL hydrochloric acid (HCl), 5 ± 10^−3^ g copper (II) chloride (CuCl_2_), 30 ± 10^−3^ mL distilled water, and 25 ± 10^−3^ mL ethanol [26]. The chemical composition of the samples was evaluated using EDS, under the same SEM conditions, with a 3 ± 10^−3^ μm beam spot.

Surface measurements were performed using a Bruker Contour GT optical profilometer (Bruker, Billerica, MA, USA). A threshold value of 5, as recommended by the default setting, was applied throughout. Measurements were acquired in Vertical Scanning Interferometry (VSI) mode using green light illumination.

## 3. Results

### 3.1. Sample Characterisation

**Figure 2:**
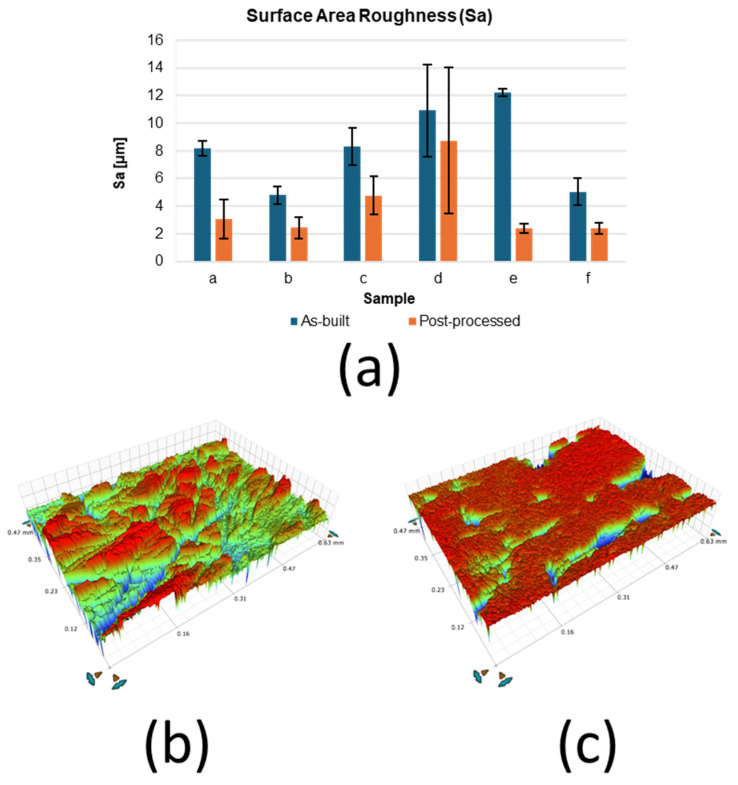
Bruker measurements: (**a**) average surface roughness Sa, surface topographies from (**b**) as-built and (**c**) finished surface.

Figure 2a presents the variations in average surface roughness (Sa) for six samples in both as-built and post-processed conditions. The results demonstrate the consistent effectiveness of centrifugal mass finishing in improving surface quality. The Sa of the sample (Figure 2a) decreased from 8.20 ± 0.54 µm in the as-built condition to 3.05 ± 1.42 µm after processing, while the sample (Figure 2b) showed the most pronounced improvement, with Sa decreasing from 12.19 ± 0.26 µm to 2.67 ± 0.33 µm. Across all samples, three hours of treatment resulted in substantial reductions in roughness, confirming the repeatability of the finishing process. This quantitative trend is further supported by the surface topography images shown in Figure 2b,c. The as-built surfaces (Figure 2b) are characterised by a rough, irregular morphology with distinct asperities, whereas the mass-finished surfaces (Figure 2c) reveal a significantly smoother and more homogeneous finish, with a notable reduction in surface asperities and enhanced uniformity.

**Figure 3:**
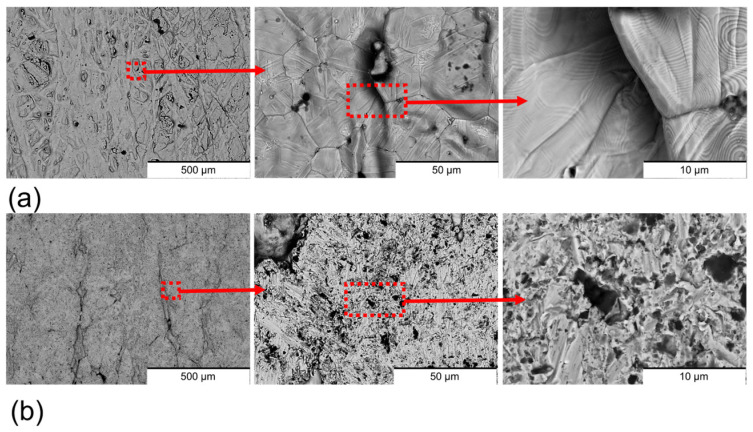
Surface BSE-SEM images of (**a**) non-polished surfaces and (**b**) polished surfaces. The zooms of the images are marked in red.

The surface morphology of the sintered 17-4PH stainless steel was significantly modified by the polishing process (Figure 3). Non-polished samples (Figure 3a) exhibited a surface characterised by polygonal cracks, which are commonly associated with inter-particle boundaries inherent to the sintering process [27,28,29]. Surface ripples confined between these defects were also present on non-polished surfaces, which can be attributed to the hydrodynamic motion of organic polymeric binders during fabrication. In addition, non-sintered powders were observed on the non-polished samples, a feature frequently reported in material extrusion additively manufactured materials [30]. By contrast, the polished samples (Figure 3b) were devoid of cracks and ripples, indicating that these imperfections were confined to the outer surface layer and removed during the polishing process. However, the non-sintered particles remained visible, suggesting that the sintering process was only partially effective throughout the bulk material. Holes and scratches with random spatial distribution were observed on the polished surfaces. These features are attributed to the nature of mechanical polishing, which involves random impacts of abrasive particles with variable peak sizes, impact force, and velocity. Depending on these parameters, the abrasive particles can generate holes or scratches during the finishing process [31].

**Figure 4:**
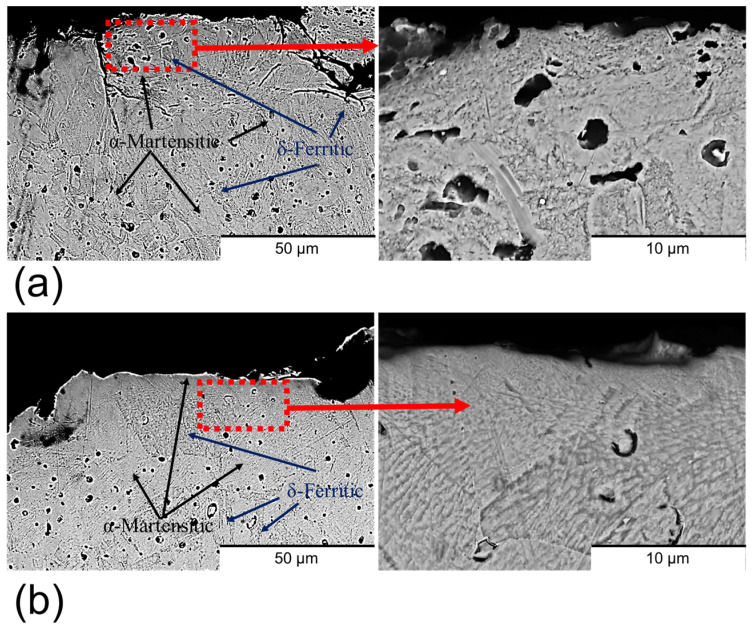
Cross-section BSE-SEM images of (**a**) non-polished surfaces and (**b**) polished surfaces. The zooms of the images are marked in red.

Cross-section imaging of the samples (Figure 4) revealed that both sample types exhibited a predominant α-martensitic microstructure with δ-ferrite phase [29,30,32]. However, differences in structural integrity were observed between the two conditions. The non-polished samples (Figure 4a) presented a high density of surface cracks, whereas these defects were absent in the polished samples (Figure 4b). This contrast is attributed to the rapid temperature fluctuations at the surface during sintering, which differ significantly from the thermal conditions within the bulk material. Such abrupt changes induce thermal stresses that are responsible for surface crack formation [33]. Importantly, the abrasive polishing removed the outer layer with cracks, containing these cracks.

**Figure 5:**
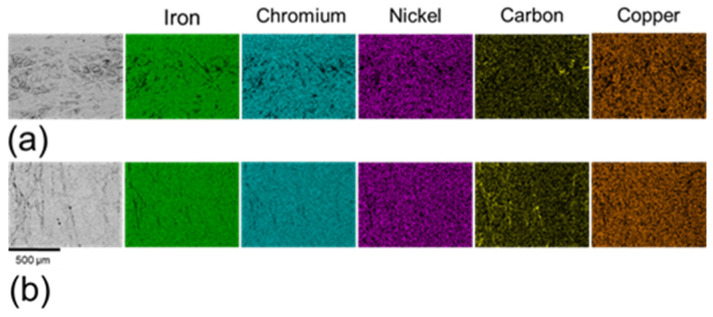
Element distribution maps of (**a**) non-polished surfaces and (**b**) polished surfaces.

As illustrated in Figure 5, most elements were homogeneously distributed across both sample types, with carbon being the notable exception. Carbon exhibited a more heterogeneous distribution in the non-polished samples (Figure 5a) compared to the polished samples (Figure 5b). The carbon observed on the non-polished surface is attributed to incomplete removal of binder materials during sintering. The sintering process typically employs filaments composed of metallic alloys bonded with organic polymers. While the majority of the organic material is eliminated through thermo-chemical reactions, incomplete sintering can lead to carbonaceous residues originating from the polymers [30,32]. These residues exhibit low adhesion to the metallic surface and can be effectively removed by the polishing process, which accounts for the reduction in areas of high carbon concentrations in polished samples.

### 3.2. Electrochemical Analyses

#### 3.2.1. AEN Analyses

**Figure 6:**
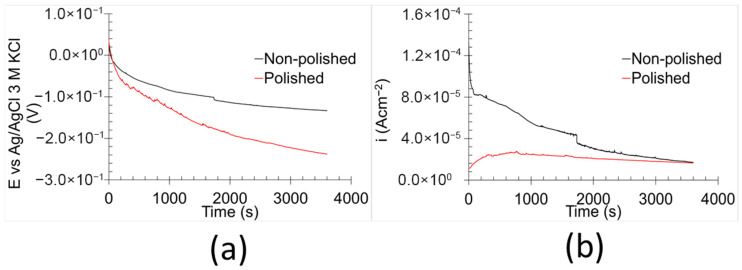
AEN results of the samples in 0.6 M NaCl at room temperature: (**a**) OCP and (**b**) ZRA.

The AEN results (Figure 6) illustrate the temporal evolution of the potential (Figure 6a) and current density (Figure 6b). Both non-polished and polished samples exhibited an exponential decrease in potential over time. However, the temporal evolution of current density differed notably between the two sample types. In the case of the non-polished samples, the current density followed a similar trend to the potential decay. This correlation suggests that the surface underwent chemical evolution without significant changes in resistance. According to Ohm’s law, resistance is directly proportional to voltage and inversely proportional to current density [34]. Thus, the analogous behaviour of both parameters implies a relatively constant resistance throughout the measured period. In contrast, the polished samples displayed a nearly constant current density over time. This phenomenon, coupled with the observed potential decay, is indicative of chemical activation of the surface [35]. However, the corrosion kinetics remained unaffected by this activation process. The observed differences in current density behaviour between non-polished and polished samples are likely due to variations in carbon distribution and surface roughness. The enhanced homogeneity of the elements in polished samples appears to promote greater corrosion stability and more consistent corrosion kinetics [30].

The corrosion features derived from the AEN data are summarised in Table 2. Equation (1) [36] was utilised to calculate the asymmetric electrochemical noise corrosion rate (*C.R._AEN_*), while Equation (2) [37,38] was used to estimate the asymmetric electrochemical noise (*R_AEN_*).

(1) $${C.R.}_{AEN}=\frac{i_{R.M.S}\times M}{n\times F\times d}$$

(2) $$R_{AEN}=\frac{\sigma_{E}}{\sigma_{i}}$$

Here, *i_R.M.S_* is the root-mean-square of the current density, *M* is the molar mass of the iron (56 gmol^−1^), *n* is the electron number transferred in the corrosion reaction (3), *F* is the Faraday constant (96,500 Cmol^−1^), and *d* is the iron density (7.8 gcm^−3^). *i_R.M.S_* was estimated using Equation (3) [39].

(3) $$i_{R.M.S}=\sqrt{\frac{\sum_{1}^{n}i_{n}^{2}}{n}}$$

where $i_{n}$ is the current density measurement for each point and n is the number of measurements.

The results show that the *C.R._AEN_* of the non-polished samples was approximately twice as high as that of the polished samples. In terms of *R_AEN_*, the polished samples displayed values an order of magnitude greater than those of their non-polished counterparts. Both parameters indicate increased corrosion performance/kinetics. Surface imperfections tend to decrease corrosion resistance by facilitating localised electrochemical activity [40,41,42]. These findings suggest that polishing enhances the kinetic corrosion resistance of 17-4PH stainless steel. However, it should be noted that AEN analyses primarily reflect cathodic reactions due to the role of the counter electrode in the setup [43].

The localisation index (L.I.) of the samples was calculated using ZRA data with Equation (4) [37,38]. L.I. values ranged between 0.10 and 1.00, reflecting the degree of localised corrosion susceptibility in the tested samples [37].

(4) $$L.I.=\frac{\sigma_{i}}{i_{R.M.S}}$$

#### 3.2.2. PPC Assessments

**Figure 7:**
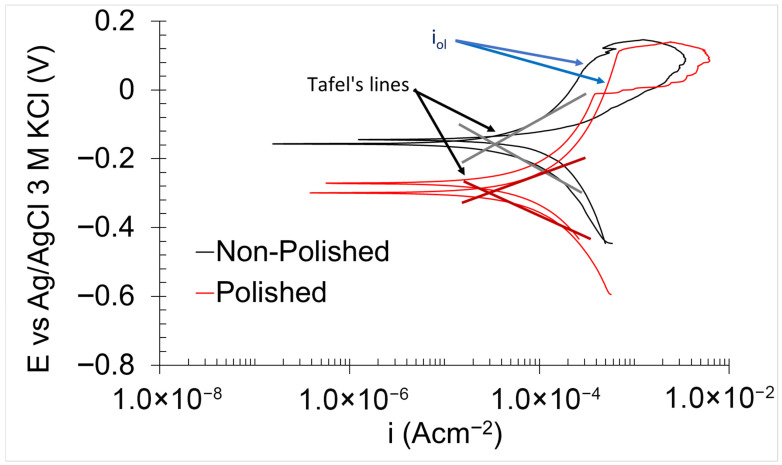
PPC for non-polished and polished 17-4PH samples in 0.6 M NaCl.

PPC obtained in 0.6 M NaCl (Figure 7) exhibited consistent behaviour across all tested samples. The cathodic branch displayed a sloped curve, which indicates a mixed control involving both activation and diffusion processes governing the reduction reactions [44]. In contrast, the anodic branch displayed a variable curve form as a function of potential. The curve exhibited a nearly vertical profile from the corrosion potential (E_corr_) to a specific potential, which is indicative of the presence of a protective oxidised layer [32,36]. However, this oxidised layer cannot be classified as a true passive film, as the corresponding current density (I_ol_) related to the oxidised layer exceeds the commonly accepted threshold of 10^−6^ Acm^−2^ [45]. The stability and protection properties of the oxidised layer for the stainless steel are strongly influenced by its chromium concentration and microstructure. The protective oxidised layer primarily consists of hydroxides of iron—chromium, which exhibits greater chemical inertness with an increase in chromium concentration. The homogeneity of the oxidised layer is also determined by the microstructure of the stainless steel. Alloys with heterogeneous microstructure generally display low corrosion resistance. Grain boundaries between two microstructure types present a highly chemically active region of the oxidised layer, facilitating the oxidised layer dissolution [46]. Notably, corrosion pits also tend to form preferentially in the martensitic microstructure [47]. Both factors contributed to elevating the current density observed in the oxidised layer. A sharp increase in current density was observed at a specific potential, known as the oxidised layer breaking potential (E_ol_), beyond which the protective capacity of the layer was completely lost [44]. Upon reverse scanning, the return curve overlapped with the forward curve at potentials higher than E_corr_, indicating that the oxidised layer possesses the ability to undergo spontaneous reformation. This recovery potential is referred to as the re-oxidised layer potential (E_rol_). Notably, this recovery capacity of the oxidised layer can also indicate a high corrosion resistance of the material under immersion/drying cycle conditions. During the immersion stage, the material undergoes damage, whereas in the subsequent drying stage, the self-healing process occurs within the oxidised layer.

PPC analyses yield critical insights into both the thermodynamic and kinetic aspects of the corrosion behaviour (Table 3).

The thermodynamic parameters include E_corr_, E_ol_, and E_rol_. E_corr_ reflects the nobility of the material, E_ol_ corresponds to the thermodynamic stability of the oxidised layer, and E_rol_ indicates the ability of the oxidised layer to undergo spontaneous regeneration [44]. As shown in Table 3, the non-polished samples exhibited higher E_corr_ values, indicating greater nobility compared to the polished surfaces. This observation can be attributed to the elevated presence of carbonaceous compounds on the non-polished surface, which generally are nobler than most metals [48]. While the thermodynamic stability of the oxidised layer (E_ol_) was comparable between the two sample types, the polished samples demonstrated a higher capacity for spontaneous regeneration than the non-polished samples. This improvement is likely due to the reduction in surface roughness, as elevated roughness can diminish the regenerative capability of the passive film and oxidised layers by hindering the formation of uniform, homogeneous protective films [6].

The kinetic parameters derived from the PPC measurements included the corrosion current density (*i_corr_*), oxidised-layer current density (*i_ol_*), corrosion rate (*C.R._corr_*), oxidised layer rate (*C.R._ol_*), and the polarisation resistance (*R_p_*). The corrosion rate (*C.R._corr_*) and oxidised layer rate (*C.R._ol_*) were calculated from i_corr_ and i_ol_ using Equations (5) and (6) [36].

(5) $${C.R.}_{corr}=\frac{i_{corr}\times M}{n\times F\times d}$$

(6) $${C.R.}_{ol}=\frac{i_{ol}\times M}{n\times F\times d}$$

*i_corr_* was calculated by Tafel line extrapolation, whereas *i_ol_* was measured from the vertical segment of the anodic branch of the PPC [44]. Both non-polished and polished samples exhibited comparable values for these parameters, indicating similar levels of kinetic corrosion resistance.

*R_p_* was determined from the PPC data using Equation (7) [49].

(7) $$R_{p}=\frac{\beta_{a}\times \beta_{c}}{2.303\times \left(\beta_{a}+\beta_{c}\right)\times i_{corr}}$$

where *β_a_* and *β_c_* are the anodic and cathodic slopes, respectively, determined from the Tafel lines [50]. The calculated *R_p_* values were comparable for both sample types, consistent with the previously discussed kinetic and thermodynamic corrosion findings.

#### 3.2.3. EIS Evaluation

**Figure 8:**
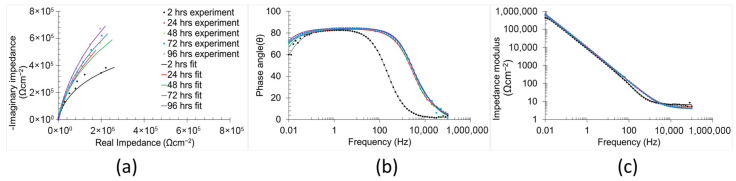
Electrochemical impedance spectra (**a**) Nyquist plots, (**b**) phase angle, and (**c**) impedance modulus Bode plots of non-polished samples in 0.6 M NaCl immersion at 2 h, 24 h, 48 h, 72 h, and 96 h.

**Figure 9:**
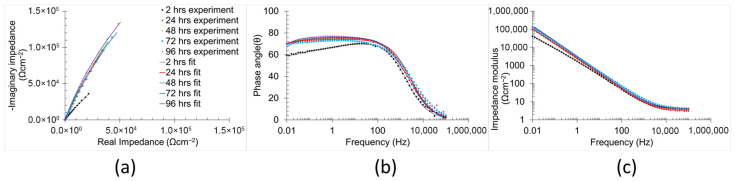
Electrochemical impedance spectra (**a**) Nyquist plots, (**b**) phase angle, and (**c**) impedance modulus Bode plots of polished samples in 0.6 M NaCl immersion at 2 h, 24 h, 48 h, 72 h, and 96 h.

The temporal evolution of the corrosion mechanisms in the samples was characterised using EIS (Figure 8 and Figure 9). Both Bode and Nyquist plots were employed to analyse the impedance data. The corrosion mechanism of the samples at different immersion times was evaluated using equivalent circuit modelling.

**Figure 10:**
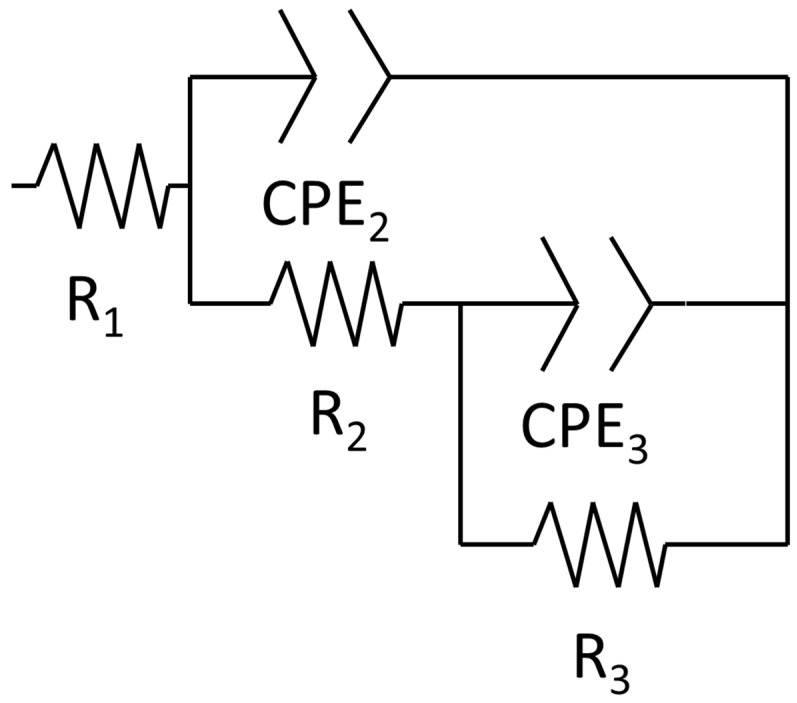
Equivalent circuit of the corrosion mechanism.

A corrosion mechanism was identified for all immersion durations and sample types. The equivalent circuit model (Figure 10) consists of a resistance in series with two time constants. The plateau curve in the Bode impedance modulus (Z_m_) versus frequency (f) plot corresponds to the series resistance (R_1_). Each time constant consisted of a resistor (R_2_ and R_3_) in parallel with a constant phase element (CPE_2_ and CPE_3_). The first time constant was associated with the medium frequency range (100 Hz–104 Hz), as evidenced by the peak in the Bode phase angle (θ) versus frequency plot, the semi-loop in the Nyquist plots, and the slope of the curve in Z_m_ versus f. The second time constant was also evident in the low frequency range (10^−1^ Hz–10^0^ Hz), indicated by the broad peak in θ versus f and the corresponding slope change in Z_m_ versus f. Notably, the constant phase element (CPE_3_) was in series with the resistor R_2_ from the preceding time constant. Identification of individual peaks for each time constant in the Bode plots (θ versus f) was complicated due to peak overlap [6,51,52].

The values of the equivalent circuit elements, corresponding to the identified corrosion mechanisms, sample types, and immersion times, are listed in Table 4. The satisfactory chi-square values (≈10^−4^) confirm the validity of the proposed equivalent circuit models in accurately representing the corrosion mechanism and their temporal evolutions according to the sample type [51,53]. Since an equivalent circuit is used in order to determine the simulated values and compare with experimental data, a CNLS (complex non-linear least squares) simulation is used, as previously reported [54]. Notably, the CNLS simulated plots can be observed in the Supplementary Data document.

R_1_ values ranged between 3 and 4 Ωcm^−2^ across all sample types and immersion durations, indicating that this resistance is associated with the solution impedance [55]. The temporal evolution of the remaining equivalent circuit element values varied according to the sample condition.

For the non-polished samples, R_2_ and CPE_2_ (first time constant) decreased (from 56.44 Ωcm^−2^ to 5.16 Ωcm^−2^) and increased (from 0.27 μFs^n−1^ cm^−2^ to 6.26 μFs^n−1^ cm^−2^), respectively, with increasing immersion duration (from 2 h to 96 h). These trends indicate a reduction in both the corrosion resistance and the thickness of the corrosion mechanism part related to this time constant. The thickness of the part is directly proportional to the value of the constant phase element (Equation (8)) [56].

(8) $$d=\frac{\varepsilon_{FeO}\times \varepsilon_{o}}{CPE}$$

Here, d is the oxidised layer thickness, $\varepsilon_{FeO}$ is the dielectric constant of the iron oxidised, and $\varepsilon_{o}$ is the permeability in the vacuum.

The low values (≈0.7) of the n_2_ observed during initial hours of immersion (2 h and 24 h) are attributed to the high density of imperfection surface and the elevated roughness of the non-polished surfaces (12.19 ± 0.26 µm). The n parameter of the constant phase element reflects the surface imperfections (e.g., pores, cracks, roughness, and voids), in which lower values indicate a high number of surface defects [57]. The n_2_ exhibited an increasing trend with the immersion duration (up to ≈0.9), suggesting a gradual smoothing process within this part of the corrosion mechanism. In contrast, the elements of the second time constant displayed the opposite trend over time (from 0.87 10^6^ Ωcm^−2^ to 3.73 10^6^ Ωcm^−2^ for R_3_, and from 23.51 μFs^n−1^ cm^−2^ to 11.21 μFs^n−1^ cm^−2^ for CPE_3_), indicating an increase in both the corrosion resistance and thickness of the corrosion mechanism part associated with this time constant. The n_3_ parameter remained stable (≈0.9) throughout the immersion period, indicating a constant level of surface imperfections in that part of the corrosion mechanism.

In respect of the polished samples, the elements associated with the first time constant exhibited different trends over time. R_2_ increased from 2 h to 48 h of immersion duration from 13.61 Ωcm^−2^ to 1220.00 Ωcm^−2^, followed by a reduction from 48 h to 96 h of immersion from 93.00 Ωcm^−2^ to 29.67 Ωcm^−2^. This behaviour suggests that, during the early stages of the immersion, aggressive samples in the harsh environment chemically react with the polished surface, promoting the formation of the protective oxidised layer that enhances the corrosion resistance [58]. However, after 48 h of immersion, the continuous exposure led to a gradual degradation of this layer, resulting in a reduction in corrosion resistance [58]. CPE_2_ and its associated exponential parameter n_2_ remained approximately stable (50–90 μFs^n−1^ cm^−2^ and ≈0.8, respectively) throughout the immersion period, indicating a roughly constant thickness [56] and surface status [57,59]. n_2_ also exhibited a higher value during the initial immersion periods in comparison with those of non-polished samples, which is attributed to its relatively low surface roughness (2.67 ± 0.33 µm) and reduced imperfection density. The elements of the second time constant remained largely unchanged across all immersion times (1–70 μFs^n−1^ cm^−2^ for CPE_3_ and 0.8–1 for n_3_), except for n_3_ at two hours of immersion, which was 0.55. This low value can be attributed to dielectric relaxation phenomena in the bulk material, which typically diminish after extended immersion [59].

#### 3.2.4. Mott-Schoktty Method Analyses

**Figure 11:**
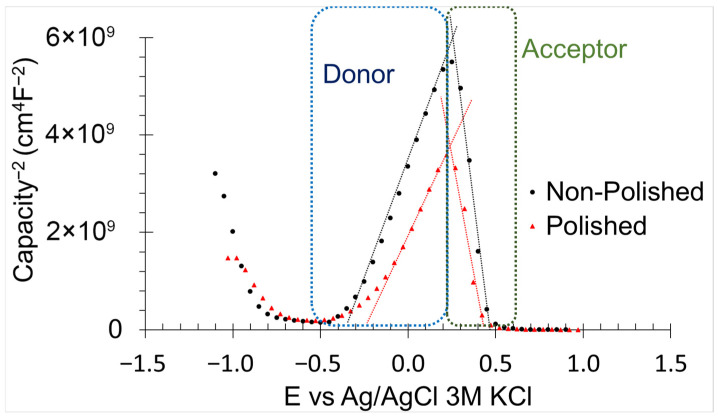
Mott-Schottky method results of non-polished and polished samples in 0.6 M NaCl immersion.

Defects within the oxidised layer of the samples, including electron donors and acceptors, were analysed using the Mott-Schoktty method (Figure 11). The electron donor density (*Nd*) and hole acceptor density (*Na*), as well as the flat band potential for donor (*E_fbd_*) and acceptor (*E_fba_*), were calculated using Equations (9) [60] and (10) [60] in conjunction with Mott-Schoktty method data.

(9) $$\frac{1}{C^{2}}=\frac{2}{Nd\times e\times \varepsilon \times \varepsilon_{o}}(E_{ap}-E_{fbd}-\frac{KT}{e})$$

(10) $$\frac{1}{C^{2}}=\frac{2}{Na\times e\times \varepsilon \times \varepsilon_{o}}(E_{ap}-E_{fba}-\frac{KT}{e})$$

where *C* is the capacitance of the samples, *e* is the electron charge (1.60 10^−19^ C), *ε_o_* is the vacuum permittivity (8.85 10^−12^ Fcm^−1^), *ε* is the iron dielectric constant (8.8), *K* is the Boltzmann constant (1.38 10^−23^ JK^−1^), and *T* is the temperature (298 K). Nd and Na were comparable between non-polished and polished samples, suggesting similar kinetic corrosion resistance (Table 5). These parameters strongly influence corrosion kinetics, as an increase in either donor or acceptor density increases the number of reactive sites, thereby accelerating corrosion [60]. Polished samples exhibited lower *E_fbd_* and higher *E_fba_* compared with non-polished samples. *E_fbd_* reflects the thermodynamic resistance to cathodic reactions, whereas *E_fba_* indicates the thermodynamic resistance to anodic reactions [60]. Surface defects, including surface roughness, cracks, pores, and voids, compromise the thermodynamic stability of the oxidised layer by creating heterogeneity in its thickness [6]. Accordingly, polishing reduces or eliminates these imperfections, resulting in a more uniform oxide layer and enhanced thermodynamic corrosion resistance.

### 3.3. Surface Analysis After Corrosion (PPC) Test

**Figure 12:**
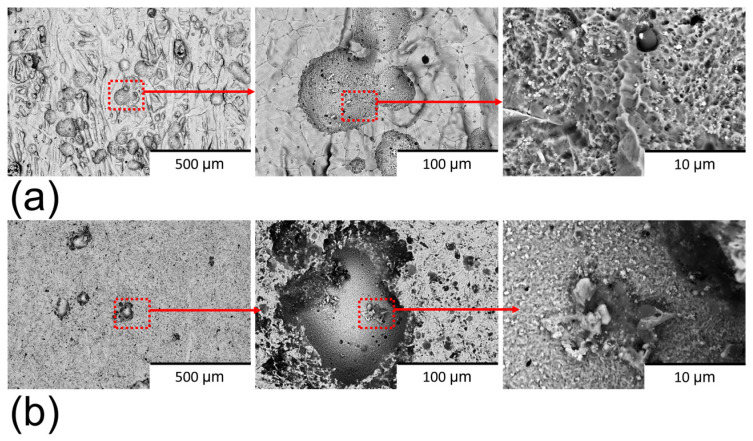
BSE-SEM images of (**a**) corroded non-polished surface and (**b**) corroded polished surface in 0.6 M NaCl.

The corroded surface of both sample types exhibited evidence of pitting, indicating the predominant localised corrosion mechanism was pitting corrosion (Figure 12). The pit number observed on corroded polished samples (Figure 12a) was lower than that on the non-polished samples (Figure 12b), suggesting superior corrosion resistance in the polished samples. On non-polished surfaces, crevices and voids act as active sites for pit formation [30]. Furthermore, the morphology of the pits varied between the two sample types. Pits on non-polished surfaces displayed a structure resembling α-martensitic appearance, which is indicative of the complete removal of the oxidised layer in those regions. Conversely, the pits on polished surfaces exhibited a smoother surface, suggesting partial reformation of the oxidised layer on the pit surface. This finding implies a higher thermodynamic corrosion resistance of the polished surface. Surface defects are known to diminish the regenerative capacity of the oxidised layer [40], and the abrasive polishing process effectively mitigates these defects, thereby contributing to improved corrosion performance of 17-4PH stainless steel.

**Figure 13:**
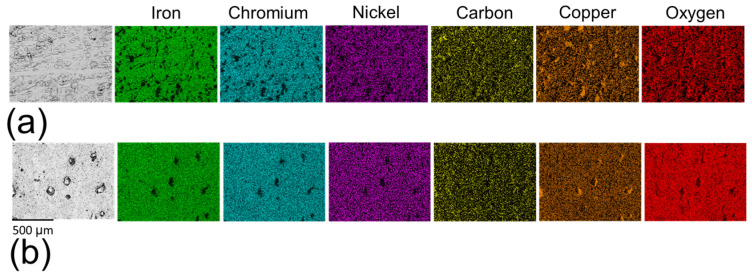
EDS mapping of (**a**) corroded non-polished surface and (**b**) corroded polished surface in 0.6 M NaCl.

The pits observed in both sample types exhibited a notable depletion of key alloy elements in 17-4PH stainless steel, including iron, chromium, and nickel (Figure 13), indicating a significant material loss due to pitting corrosion. A high concentration of copper was detected within the pit regions, suggesting in situ formation of ε-copper precipitates [27,32]. These precipitates can exacerbate the corrosion due to their cathodic effect relative to the δ-ferrite [61]. The pit regions also demonstrated a reduction in oxygen concentration compared to non-corroded regions, reflecting degradation of the oxidised layer. This reduction was more pronounced in non-polished samples (Figure 13a) than in polished surfaces (Figure 13b), indicating the greater thermodynamic stability of the oxidised layer on the polished samples. Notably, some pits on a non-polished surface also exhibited an elevated concentration of carbon, which can further facilitate the pitting corrosion formation through its cathodic effect on the matrix [48]. These observations underscore the critical influence of surface conditions in influencing on the chemical stability and corrosion resistance of the 17-4PH stainless steel.

## 4. Discussion

The results presented in Figure 2a clearly demonstrate the effectiveness of mass finishing in reducing the surface roughness of AM 17-4PH stainless steel. Across all six samples, the average surface roughness (Sa) decreased significantly after three hours of processing, highlighting the consistency and reliability of the surface treatment. The most significant improvement was observed in sample (e), where Sa was reduced by nearly 80%, from 12.19 (±0.26) µm to 2.67 (±0.33) µm. These findings are supported by the surface morphology images in Figure 2b,c, which reveal the transformation from irregular, asperity-rich topographies in the as-built condition to smoother and more homogeneous surfaces after the finishing process. The reduction in asperities is particularly significant, as these features are known to act as preferential sites for crack initiation, stress concentration, and localised corrosion.

Comparable results have been reported in studies on other alloys. For Ti-6Al-4V, mechanical surface finishing significantly reduced roughness and improved fatigue life [14]. Similarly, for Inconel 718, ultrasonic nanocrystal surface modification reduced roughness and enhanced fatigue performance [62]. Furthermore, Opoz et al. [12] demonstrated that centrifugal disc finishing not only enhanced the isotropy but also improved the tensile properties of 17-4PH stainless steel, which further confirms the potential of this approach for AM components. The consistency of these findings across different alloy systems suggests that the mechanism of improvement is broadly applicable. In the context of 17-4PH stainless steel, the noticeable reduction in surface roughness observed in the present study strongly suggests that centrifugal mass finishing can mitigate the detrimental surface features introduced during AM fabrication, thereby enhancing both corrosion resistance and structural integrity.

Higher E_rol_ (Section 3.2.2) and nobler temporal potential (Section 3.2.1) of the polished samples indicate superior thermodynamic corrosion resistance compared with the non-polished surfaces. The open circuit potential reflects the nobility of the metallic material [35], while a broader range of E_rol_—E_corr_ indicates an increased likelihood of spontaneous recovery of the oxidised layer [44]. The higher thermodynamic stability of the polished surface is attributed to the elevated E_fbd_ and E_fba_ within the oxidised layer (Section 3.2.4). These parameters are directly associated with the thermodynamic feasibility of the redox reactions. An increase in the E_fbd_—E_corr_ and E_fba_—E_corr_ ranges signifies an enhancement in the thermodynamic corrosion resistance for the samples for both oxidation and reduction processes [61]. The reduced thermodynamic stability observed in non-polished surfaces is associated with surface defects such as surface roughness, cracks, and voids, which promote crevice corrosion and microgalvanic cell formation due to the heterogeneous distribution of alloying elements [30,41,42]. All of these defects were reduced by the abrasive polishing process, thereby enhancing the thermodynamic resistance of the surfaces.

The proposed equivalent circuit corresponds to the corrosion mechanism identified. The equivalent circuit element was associated with a specific component of these corrosion mechanisms. For the first corrosion mechanism, R_1_ was related to the solution resistance (R_s_), as it remained relatively constant throughout the distinct immersion periods [55]. The first time constant was linked to the oxidised layer, where CPE_2_ corresponds to the double layer formed by the charge alignment between the solution and oxidised layer (CPE_ol_) [48]. R_2_ represented the oxidised layer resistance of the charge transference (R_ol_) [48]. The second constant time described the double layer (CPE_bar_) and charge transfer resistance (R_ba_) for the bare material.

**Figure 14:**
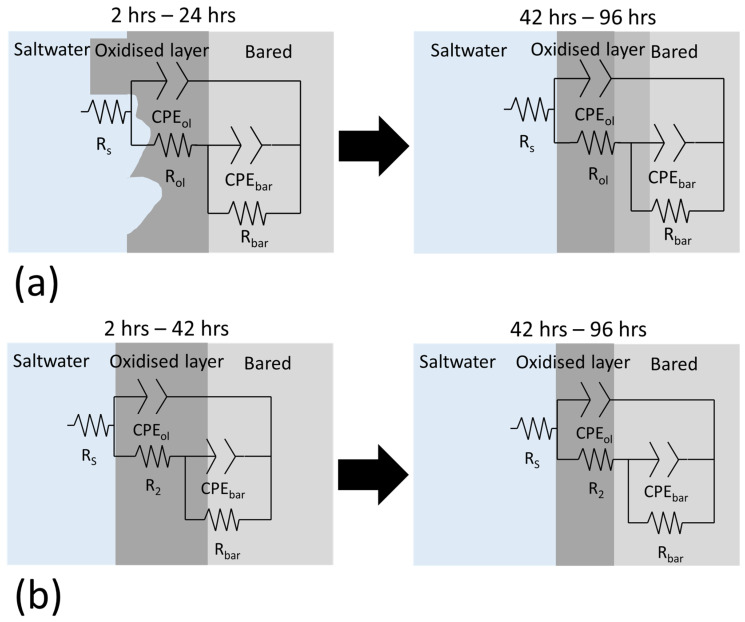
Temporal evolution of the corrosion mechanisms of (**a**) non-polished and (**b**) polished surfaces in 0.6 M NaCl.

The temporal evolution of the corrosion mechanism varied according to sample types (Figure 14). In non-polished samples, the corrosion mechanism (Figure 14a) exhibited a reduction in oxidised layer resistance and thickness, indicating the progressive dissolution. Notably, the increase in CPE_ol_ indicates the oxidised layer decrease, whereas the reduction in R_ol_ corresponds to the corrosion resistance reduction. The surface defects chemically activate the surfaces by the creation of a local site with high electrochemical activity, thereby promoting the dissolution and degradation of the oxidised layer [15]. This continuous degradation contributed significantly to a reduction in the corrosion resistance of the non-polished samples over time. The increase in R_ba_ (R_3_) also supported this assertion, as it indicates the occurrence of internal corrosion within the material. In contrast, for the polished samples (Figure 14b), R_ol_ (R_2_) exhibited an initial increase with time in the early immersion periods (2–48 h), which is attributed to the chemical evolution of the oxidised layer into a more stable and inert form. However, after 72 h of immersion, R_ol_ began to decrease, likely due to further chemical evolution of the oxidised layer, which rendered it more active and susceptible to dissolution.

Notably, the dissolution of the oxidised layer is attributed to the chemical reaction between chloride ions and iron oxides, as illustrated in Equations (11) and (12) [15].

(11) $$FeO+H_{2}O+2{Cl}^{-}\to Fe{Cl}_{2}+{OH}^{-}$$

(12) $${Fe}_{2}O_{3}+{3H}_{2}O+6{Cl}^{-}\to 2Fe{Cl}_{3}+{6OH}^{-}$$

In this study, polished MBFF-fabricated samples exhibited enhanced oxidised layer stability and reduced pitting compared with non-polished surfaces. These observations are consistent with the qualitative trends reported by Naim et al. [16], who found that post-processing treatments such as polishing reduce surface roughness and improve corrosion resistance in MBFF components. Similarly, Świetlicki et al. [17] reported that, while shot peening can increase surface roughness and locally decrease corrosion resistance in DMLS-fabricated 17-4PH, subsequent heat treatment enhances overall electrochemical stability, emphasising the importance of surface condition and microstructural refinement. In LPBF-fabricated 17-4PH, Sabooni et al. [18] observed that heat treatment homogenises the microstructure and reduces porosity, resulting in improved passive layer performance and lower pitting susceptibility. Martins et al. [19] also reported that mechanical finishing and polishing of AM-repaired steel mitigates localised corrosion and limits microgalvanic activity. These qualitative comparisons indicate a general trend whereby post-processing that reduces surface defects and optimises microstructure consistently enhances passive layer stability and decreases localised corrosion in AM 17-4PH stainless steel.

## 5. Conclusions

This study provides valuable insights into the influence of abrasive polishing on the corrosion behaviour of 17-4PH stainless steel. The principal conclusions derived from this investigation are summarised below.

- Polished samples demonstrate a higher thermodynamic corrosion resistance and enhanced capacity of spontaneous regeneration of the oxidised layer (−0.297 V and 0 V obtained from PPC) compared to non-polished surfaces. This improvement is attributed to the abrasive polishing process, which reduces the surface imperfections, including roughness, chemical heterogeneity, and cracks. These defects significantly affect the stability of the oxidised layer (Flat band potential range from −0.27 V to 0.31 V for non-polished, and from −2.97 V to 0.42 V for polished, values obtained from Mott-Schottky).
- The temporal evolution of the corrosion mechanism was strongly influenced by the surface condition. Non-polished samples displayed a continuous degradation of the oxidised layer (from 56.44 Ωcm^−2^ to 5.16 Ωcm^−2^ obtained from EIS). The high surface roughness promotes microgalvanic cell formation, which enhances the chemical reactivity of the oxidised layer and its dissolution. In contrast, polished surfaces exhibited no diffusion-controlled processes over time. For immersion periods shorter than 48 h, the corrosion resistance of the oxidised layer increased progressively (from 13.61 Ωcm^−2^ to 1220.00 Ωcm^−2^ obtained from EIS) with the immersion time.
- The pitting corrosion was identified as the dominant localised corrosion type for both sample types (0.52 and 0.15 L.I. calculated from AEN for non-polished and polished). The pits contained ε-copper precipitate with high copper concentration, accelerating the corrosion rate through its cathodic effect on the iron matrix. The polished surface exhibited a lower number of pits compared to the non-polished surface, indicating an improvement in the local corrosion resistance.

Overall, this study demonstrates that the abrasive polishing process significantly enhances both the thermodynamic behaviour and localised corrosion resistance of sintered 17-4 PH stainless steel. These findings have important implications for the optimisation of post-processing strategies in additive manufacturing and provide a better understanding of how surface conditions influence the corrosion behaviour of the sintered 17-4PH stainless steel, addressing a previously underexplored gap in the field. Additionally, the effect of the other treatment, such as heat treatment, on the polished samples will be examined in future studies.

## Figures and Tables

**Table 1:** Chemical composition of 17-4PH stainless steel in wt.%.

	Cr	Ni	Cu	Si	Mn	Nb	C	P	S	Fe
Min–max	15.00–17.50	35.00	35.00	1.00	1.00	0.15–0.45	0.07	0.04	0.03	Balance

**Table 2:** Corrosion characteristics obtained from AEN analyses of the samples.

Sample	*i_R.M.S_* (Acm^−2^)	*σ_i_* (Acm^−2^)	*C.R._AEN_* (μms^−1^)	*L.I.*	*σ_E_* (V)	*R_AEN_* (Ωcm^2^)
Non-polished	4.3 × 10^−5^ ± 0.3 × 10^−5^	2.2 × 10^−5^ ± 0.1 × 10^−5^	329 ± 3	0.52 ± 0.02	0.032 ± 0.001	1.44 × 10^3^ ± 0.03 × 10^3^
Polished	2.1 × 10^−5^ ± 0.2 × 10^−5^	0.32 × 10^−5^ ± 0.01 × 10^−5^	162 ± 4	0.15 ± 0.01	0.062 ± 0.02	18.96 × 10^3^ ± 0.05 × 10^3^

**Table 3:** Thermodynamic and kinetic characteristics of the samples in 0.6 M NaCl obtained using PPC.

Sample	E_corr_ (V)	E_ol_ (V)	i_corr_ (Acm^−2^)	β_c_	β_a_	*R_p_* (Ωcm^2^)	i_ol_ (Acm^−2^)	*C.R._corr_* (µm/Year)	*C.R._ol_* (µm/Year)	E_rol_ (V)
Non-polished	−0.16 ± 0.01	0.10 ± 0.01	3.8 × 10^−5^ ± 0.2 × 10^−5^	−0.075 ± 0.005	0.072 ± 0.006	2.3 × 10^4^ ± 0.2 × 10^4^	3.8 × 10^−4^ ± 0.2 × 10^−4^	293 ± 1	2.9 × 10^3^ ± 0.1 × 10^3^	−0.14 ± 0.01
Polished	−0.30 ± 0.03	0.11 ± 0.01	4.6 × 10^−5^ ± 0.2 × 10^−5^	−0.072 ± 0.005	0.074 ± 0.004	2.6 × 10^4^ ± 0.2 × 10^4^	4.1 × 10^−4^ ± 0.3 × 10^−5^	352 ± 1	3.2 × 10^3^ ± 0.2 × 10^3^	−0.010 ± 0.001

**Table 4:** Equivalent circuit characteristics of the samples in immersion in 0.6 M NaCl at varying times.

Sample	Immersion Time (h)	R_1_ (Ωcm^2^)	R_2_ (Ωcm^2^)	CPE_2_ (μFs^n−1^ cm^−2^)	n_2_	R_3_ (Ωcm^2^)	CPE_3_ (μFs^n−1^ cm^−2^)	n_3_	χ^2^ (10^−4^)
Non-polished	2	4.55	56.44	0.27	0.71	0.87 × 10^6^	23.51	0.93	9.40
	24	4.61	45.00	0.18	0.73	1.10 × 10^6^	18.72	0.94	10.68
	48	4.10	7.24	6.37	0.99	2.35 × 10^6^	12.21	0.89	7.62
	72	4.32	5.19	6.29	0.98	2.98 × 10^6^	11.90	0.89	5.84
	96	4.13	5.16	6.26	0.98	3.73 × 10^6^	11.21	0.90	4.70
Polished	2	4.31	13.61	87.10	0.84	0.87 × 10^6^	72.03	0.55	4.61
	24	3.33	1220.00	90.91	0.83	1.47 × 10^6^	1.92	0.80	8.90
	48	3.80	93.00	82.62	0.83	1.31 × 10^6^	2.66	1.00	4.90
	72	4.31	30.69	51.63	0.86	0.99 × 10^6^	24.80	0.80	3.24
	96	4.26	29.67	51.82	0.86	1.12 × 10^6^	18.73	0.84	2.61

**Table 5:** Characteristics of the defects in the oxidised layer of the samples obtained by Mott-Schoktty method.

Sample	*Nd* (Electron/cm^2^)	*E_fbd_* (V)	*Na* (Holes/cm^2^)	*E_fba_* (V)
Non-polished	1.76 × 10^21^	−0.27	5.88 × 10^20^	0.31
Polished	2.94 × 10^21^	−2.97	8.83 × 10^20^	0.42

## Data Availability

The data are in the manuscript and Supplementary Data document.

## Supplementary Materials

The following supporting information can be downloaded at: https://www.mdpi.com/article/10.3390/ma18225148/s1, Supplementary Data document.
