# Supplementary material for: An Electrochemical Study of the Corrosion Behaviour of the Polished Atomic Diffusion Additive Manufactured 17-4PH Stainless Steel Using Centrifugal Mass Finishing Method in Saltwater
Source: Materials (Basel). 2025 Nov 12;18(22):5148. doi: 10.3390/ma18225148 (PMC12654127; doi:10.3390/ma18225148)
Supplement: Supplementary file 1 [file materials-18-05148-s001.zip › materials-3909760-supplementary.pdf]

Supplementary data

Open circuit potential (duplicity)

Non-polished 17-4PH

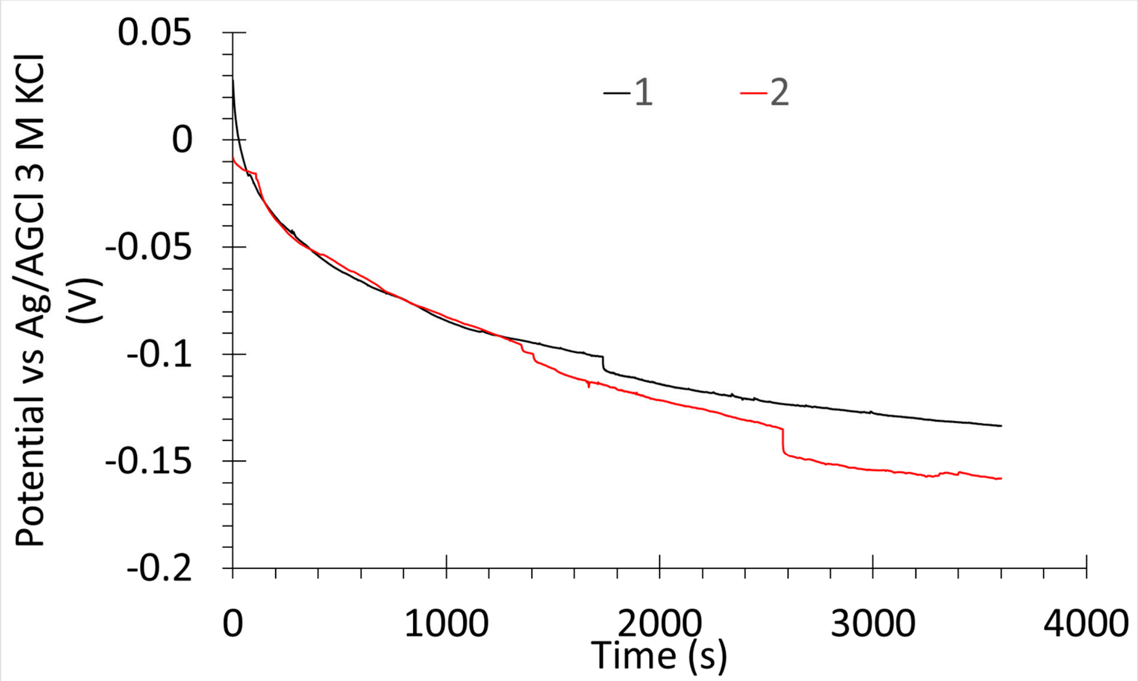

Polished 17-4PH

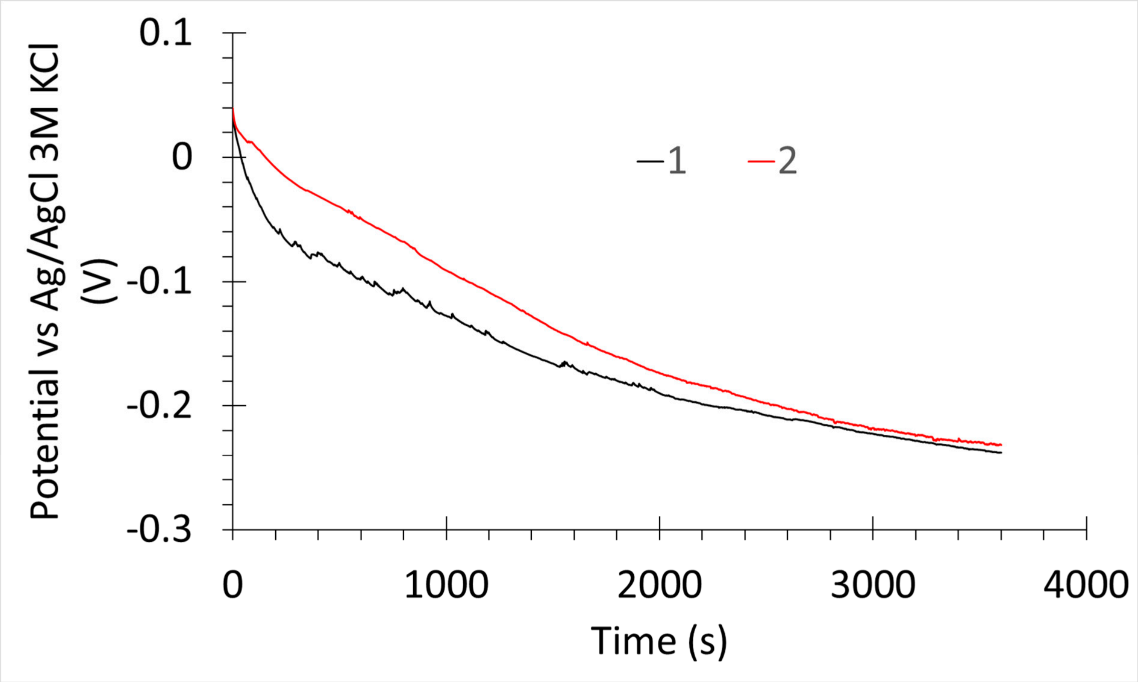

Zero Resistance Ammeter (duplicity)

Non-polished 17-4PH

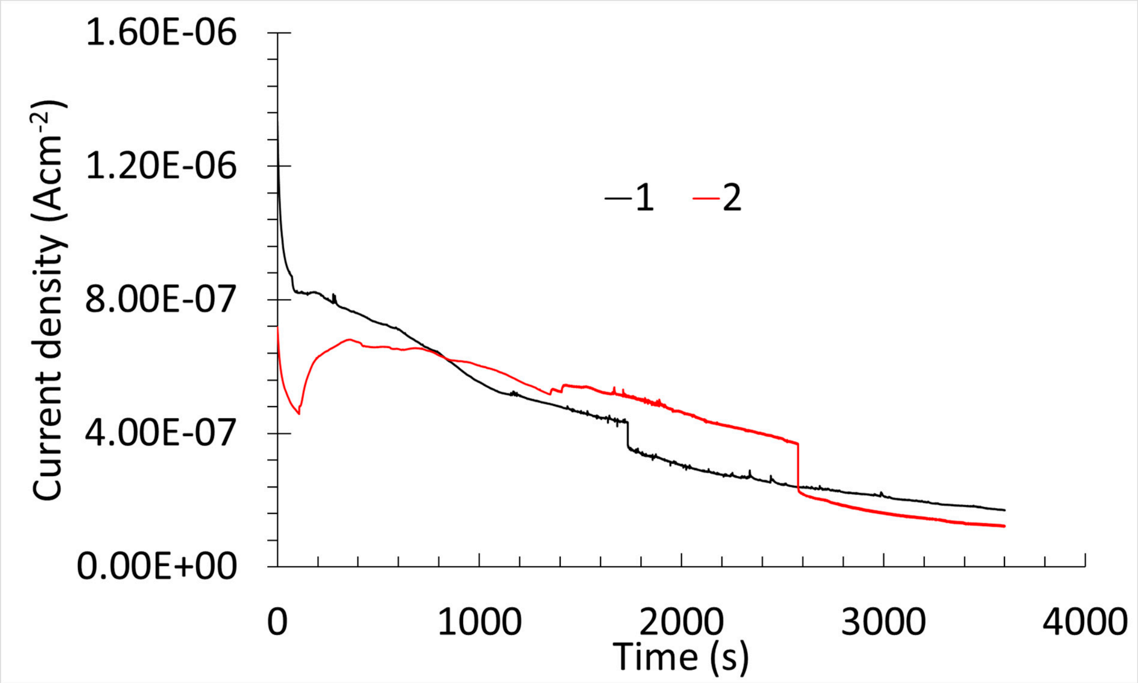

Polished 17-4PH

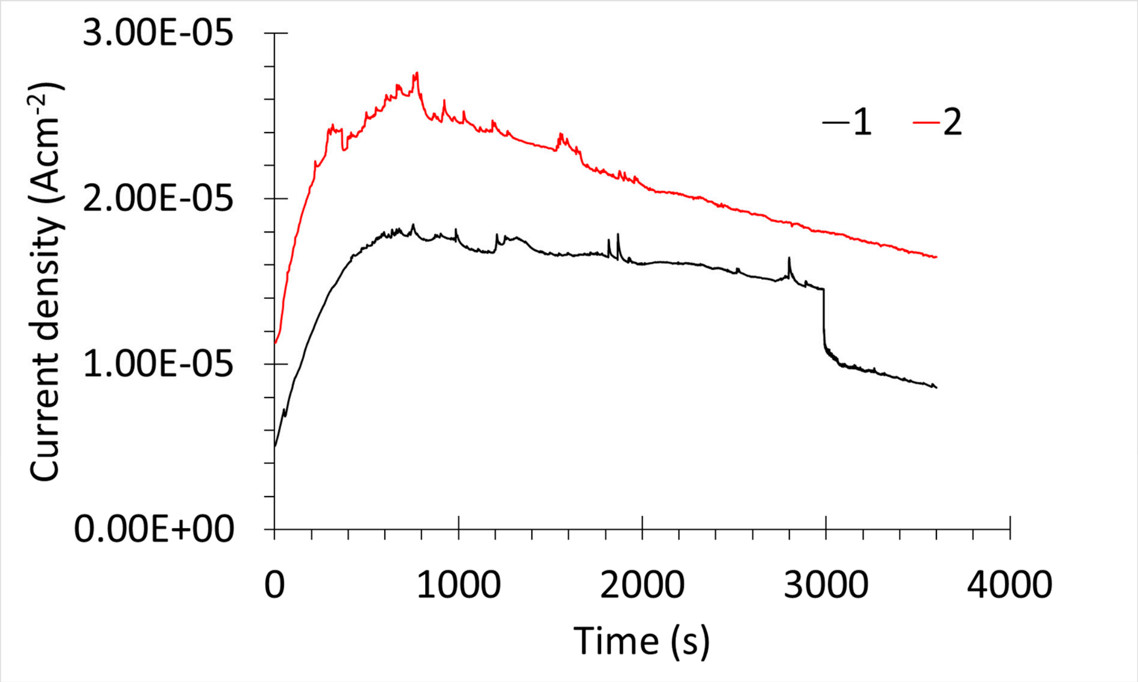

Electrochemical impedance spectroscopy (Experimental and simulated plots)

Non-polished

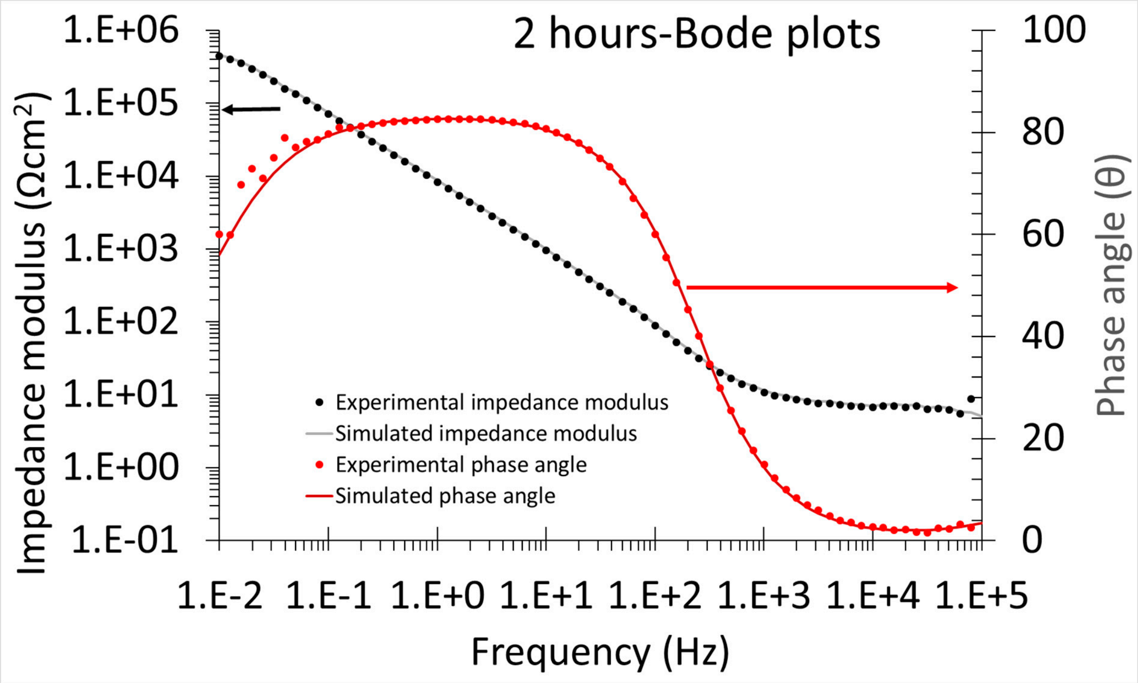

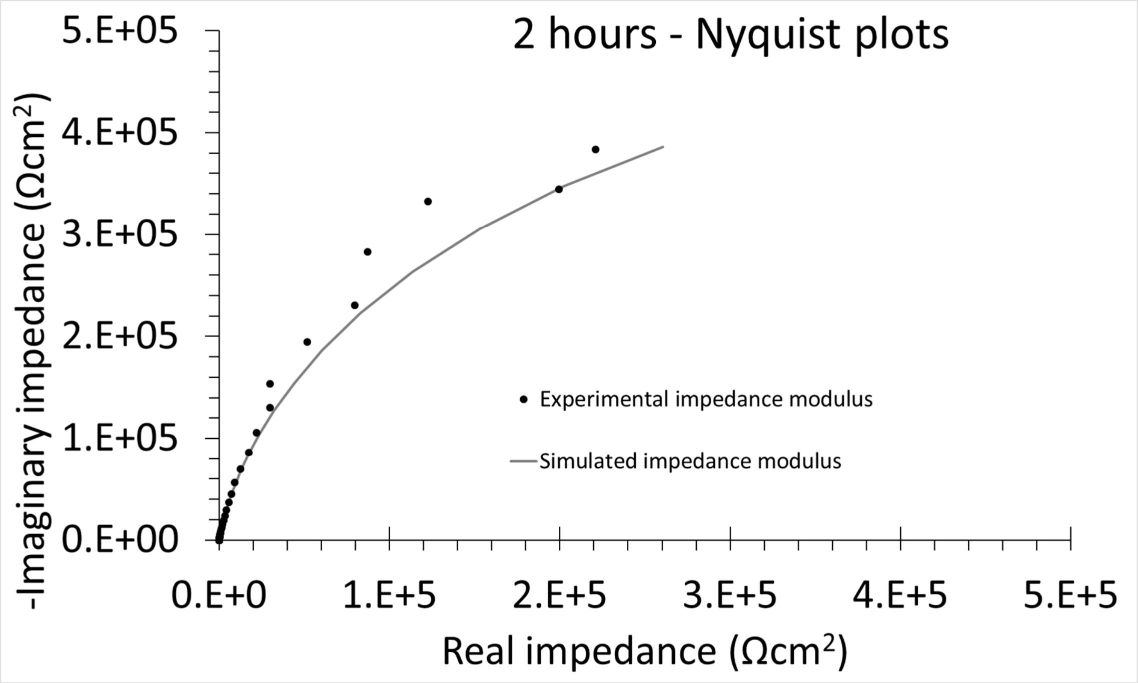

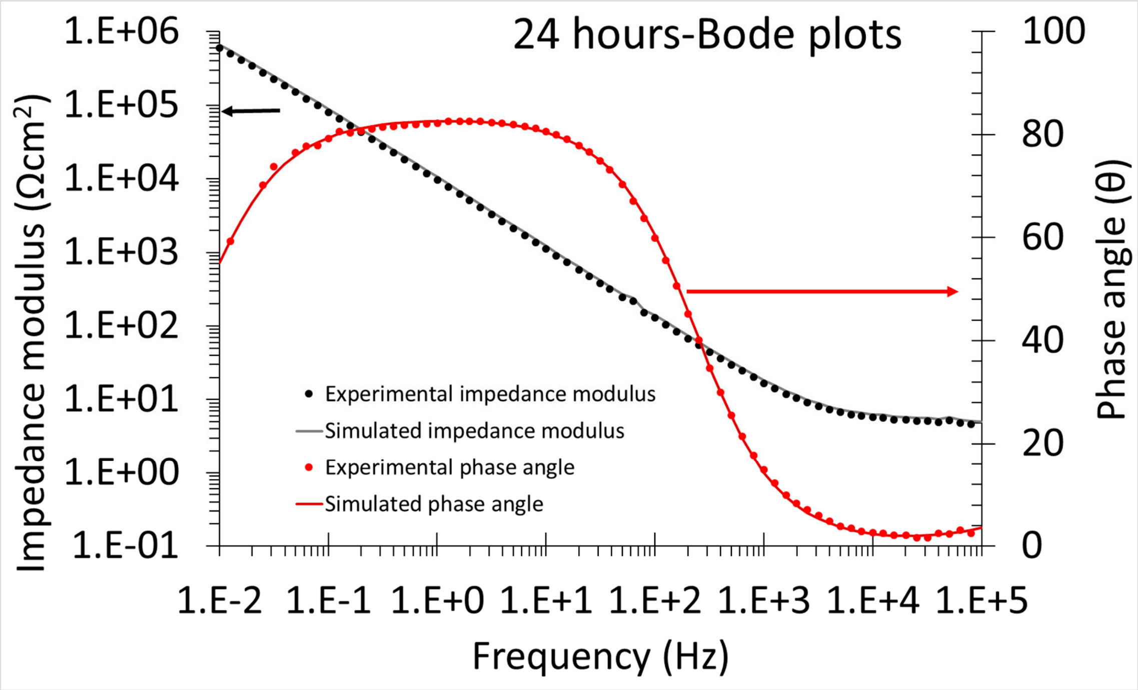
24 hours-Bode plots
Impedance modulus (Ωcm²)
Phase angle (θ)
Frequency (Hz)
1.E+06
1.E+05
1.E+04
1.E+03
1.E+02
1.E+01
1.E+00
1.E-01
100
80
60
40
20
0
1.E-2
1.E-1
1.E+0
1.E+1
1.E+2
1.E+3
1.E+4
1.E+5
Experimental impedance modulus
Simulated impedance modulus
Experimental phase angle
Simulated phase angle

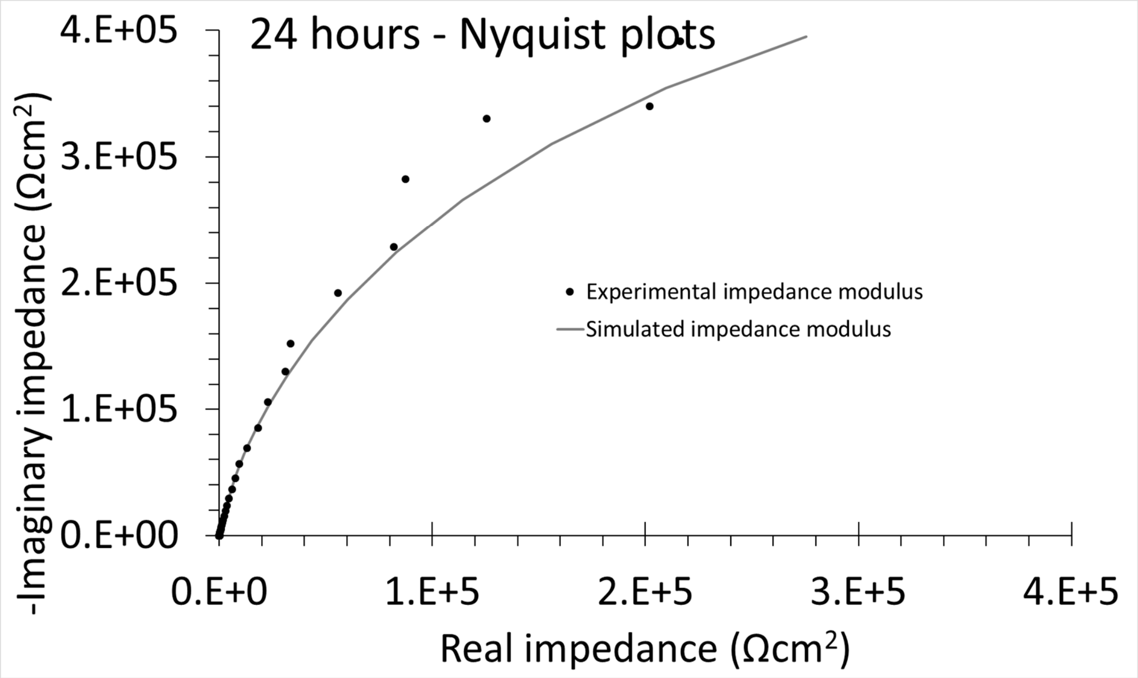
24 hours - Nyquist plots
-Imaginary impedance (Ωcm²)
Real impedance (Ωcm²)
4.E+05
3.E+05
2.E+05
1.E+05
0.E+00
0.E+0
1.E+5
2.E+5
3.E+5
4.E+5
Experimental impedance modulus
Simulated impedance modulus

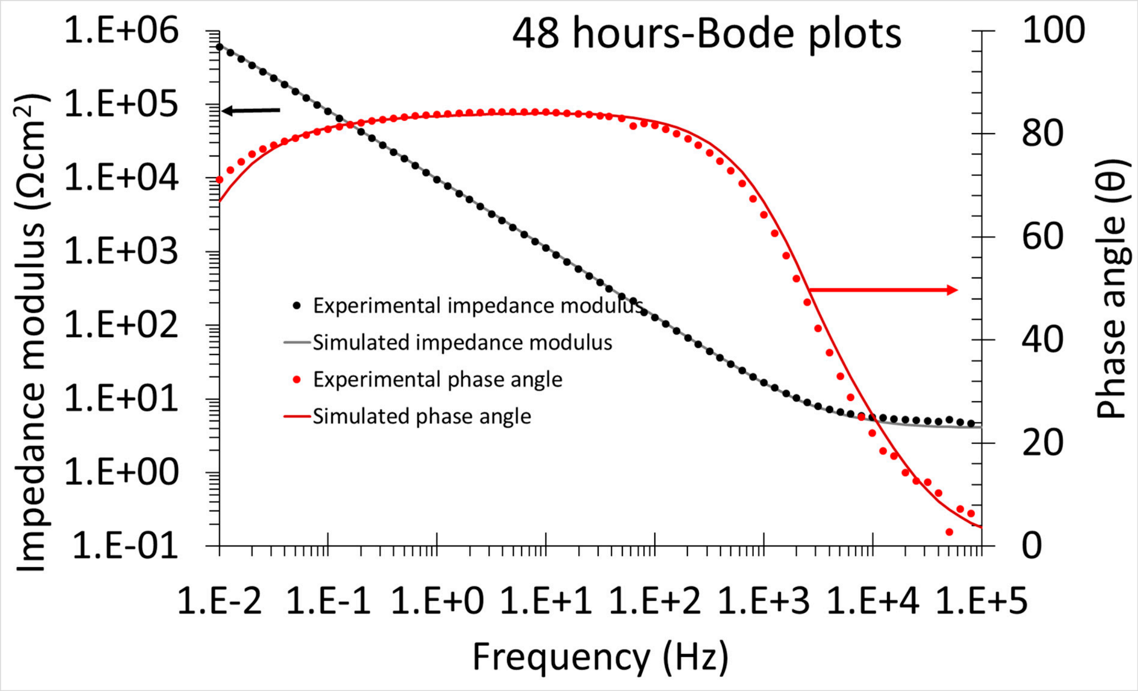
48 hours-Bode plots
Impedance modulus (Ωcm2)
Phase angle (θ)
Frequency (Hz)
1.E+06
1.E+05
1.E+04
1.E+03
1.E+02
1.E+01
1.E+00
1.E-01
100
80
60
40
20
0
1.E-2
1.E-1
1.E+0
1.E+1
1.E+2
1.E+3
1.E+4
1.E+5
Experimental impedance modulus
Simulated impedance modulus
Experimental phase angle
Simulated phase angle

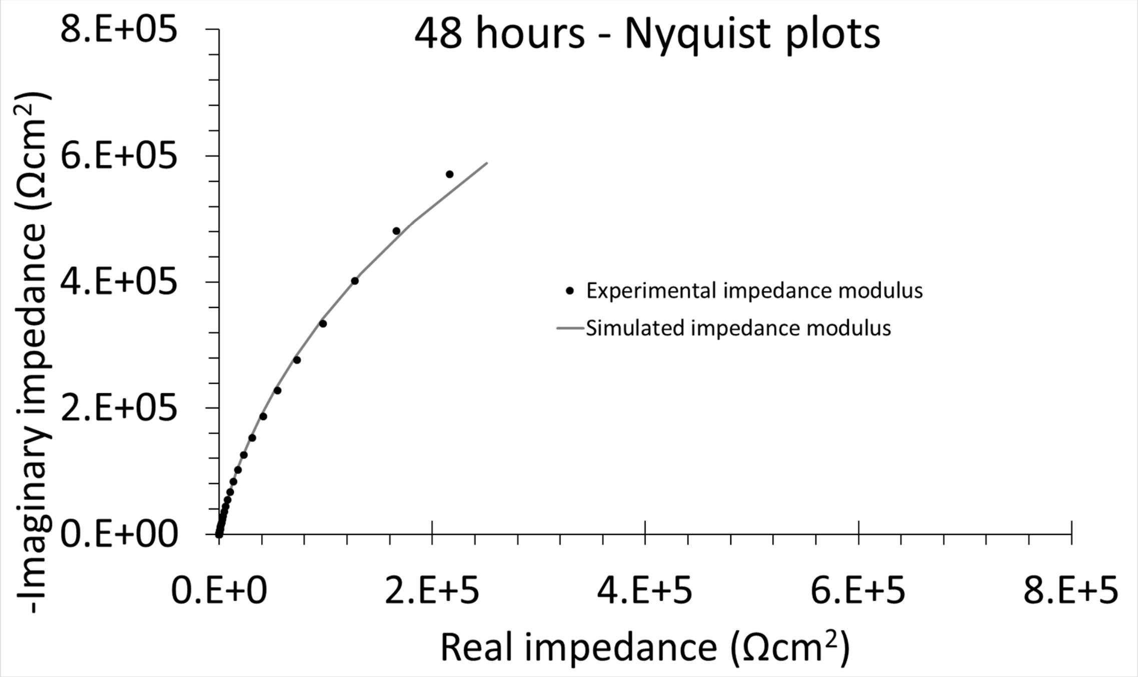
48 hours - Nyquist plots
-Imaginary impedance (Ωcm2)
Real impedance (Ωcm2)
8.E+05
6.E+05
4.E+05
2.E+05
0.E+00
0.E+0
2.E+5
4.E+5
6.E+5
8.E+5
Experimental impedance modulus
Simulated impedance modulus

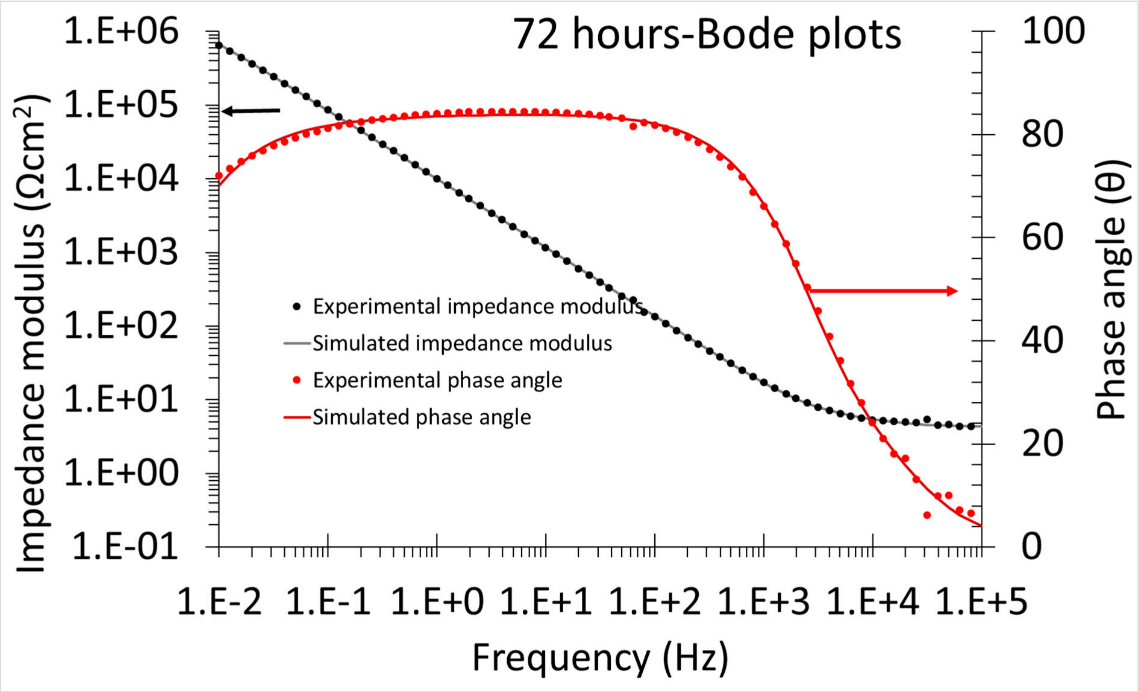

72 hours-Bode plots
Impedance modulus (Ωcm2)
Phase angle (θ)
Frequency (Hz)
1.E+06
1.E+05
1.E+04
1.E+03
1.E+02
1.E+01
1.E+00
1.E-01
100
80
60
40
20
0
1.E-2
1.E-1
1.E+0
1.E+1
1.E+2
1.E+3
1.E+4
1.E+5
Experimental impedance modulus
Simulated impedance modulus
Experimental phase angle
Simulated phase angle

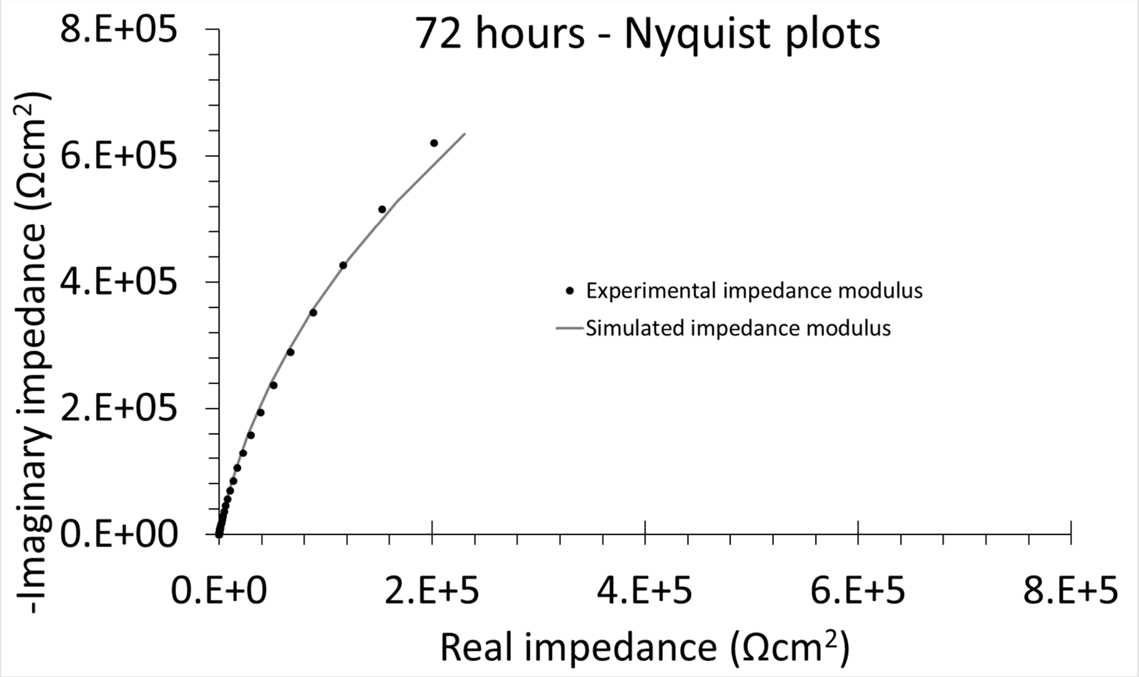

72 hours - Nyquist plots
-Imaginary impedance (Ωcm2)
Real impedance (Ωcm2)
8.E+05
6.E+05
4.E+05
2.E+05
0.E+00
0.E+0
2.E+5
4.E+5
6.E+5
8.E+5
Experimental impedance modulus
Simulated impedance modulus

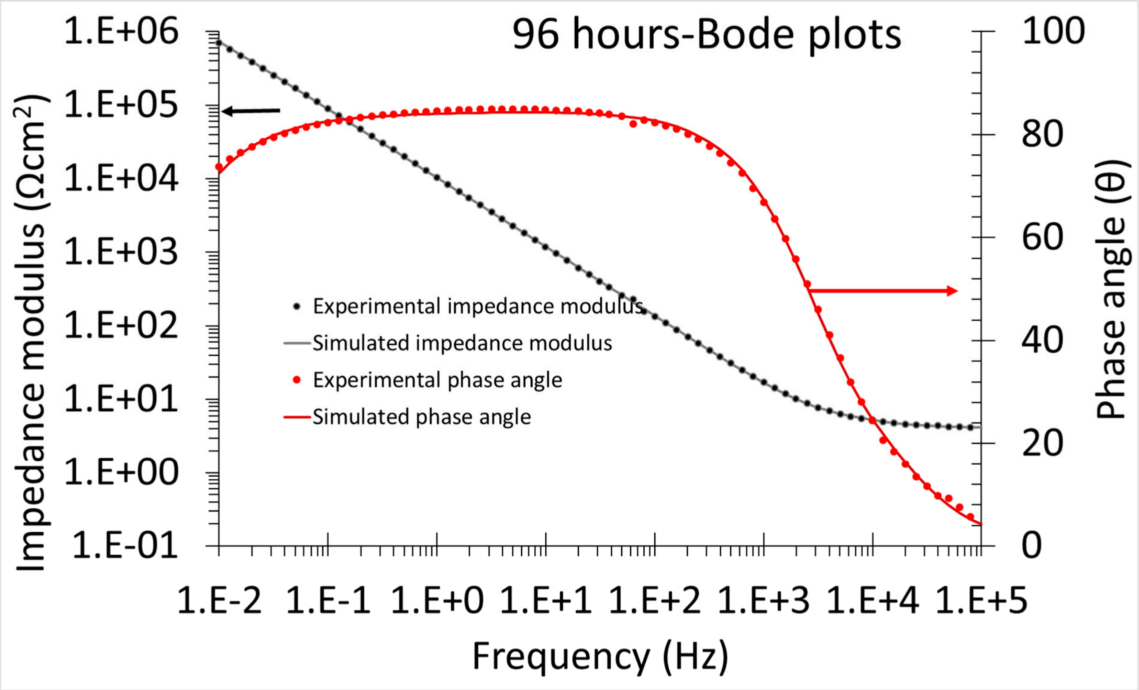
96 hours-Bode plots
Impedance modulus (Ωcm2)
Phase angle (θ)
Frequency (Hz)
1.E+06
1.E+05
1.E+04
1.E+03
1.E+02
1.E+01
1.E+00
1.E-01
100
80
60
40
20
0
1.E-2
1.E-1
1.E+0
1.E+1
1.E+2
1.E+3
1.E+4
1.E+5
Experimental impedance modulus
Simulated impedance modulus
Experimental phase angle
Simulated phase angle

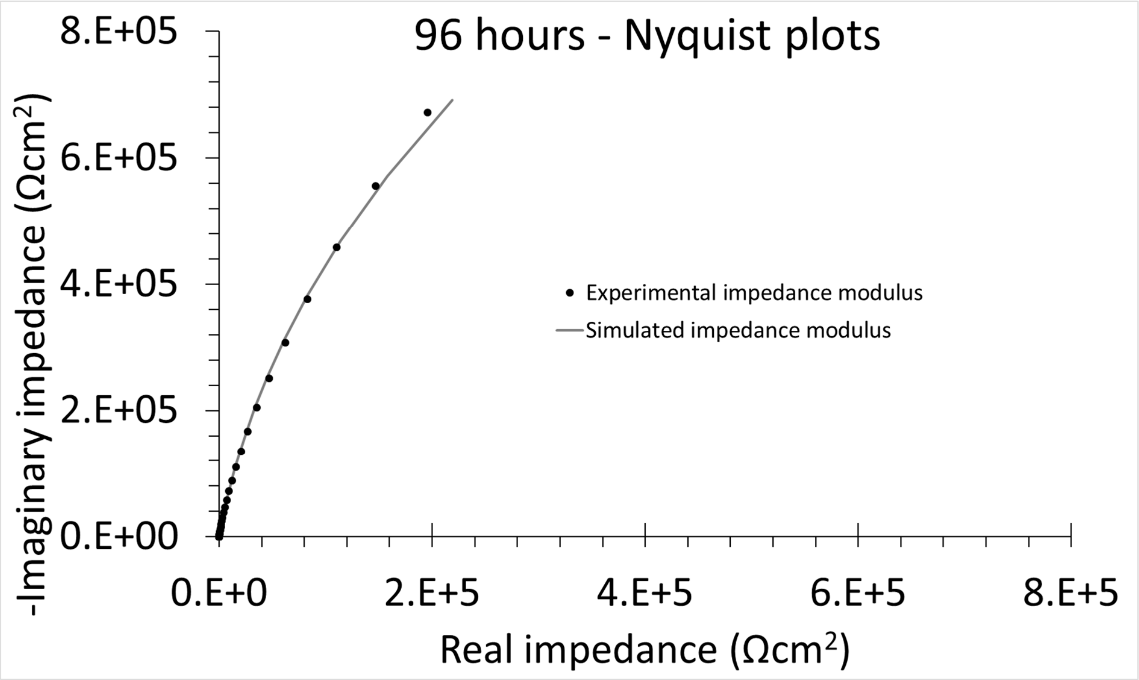
96 hours - Nyquist plots
-Imaginary impedance (Ωcm2)
Real impedance (Ωcm2)
8.E+05
6.E+05
4.E+05
2.E+05
0.E+00
0.E+0
2.E+5
4.E+5
6.E+5
8.E+5
Experimental impedance modulus
Simulated impedance modulus

Polished

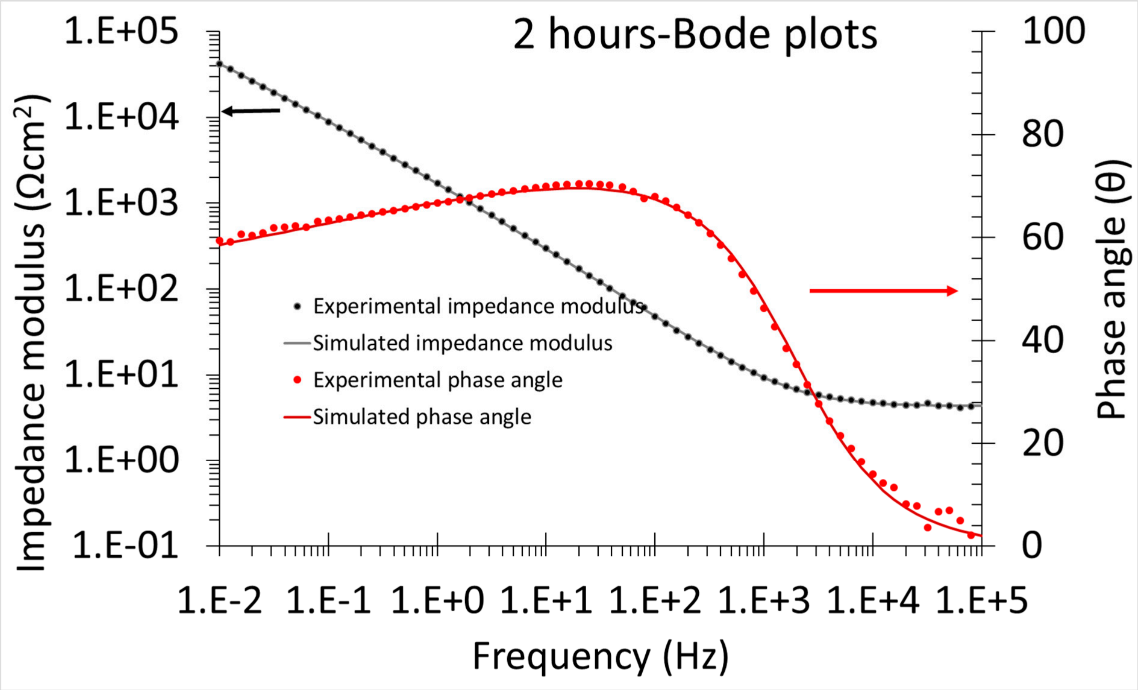
2 hours-Bode plots
Impedance modulus (Ωcm2)
Phase angle (θ)
Frequency (Hz)
1.E+05
1.E+04
1.E+03
1.E+02
1.E+01
1.E+00
1.E-01
100
80
60
40
20
0
1.E-2
1.E-1
1.E+0
1.E+1
1.E+2
1.E+3
1.E+4
1.E+5
Experimental impedance modulus
Simulated impedance modulus
Experimental phase angle
Simulated phase angle

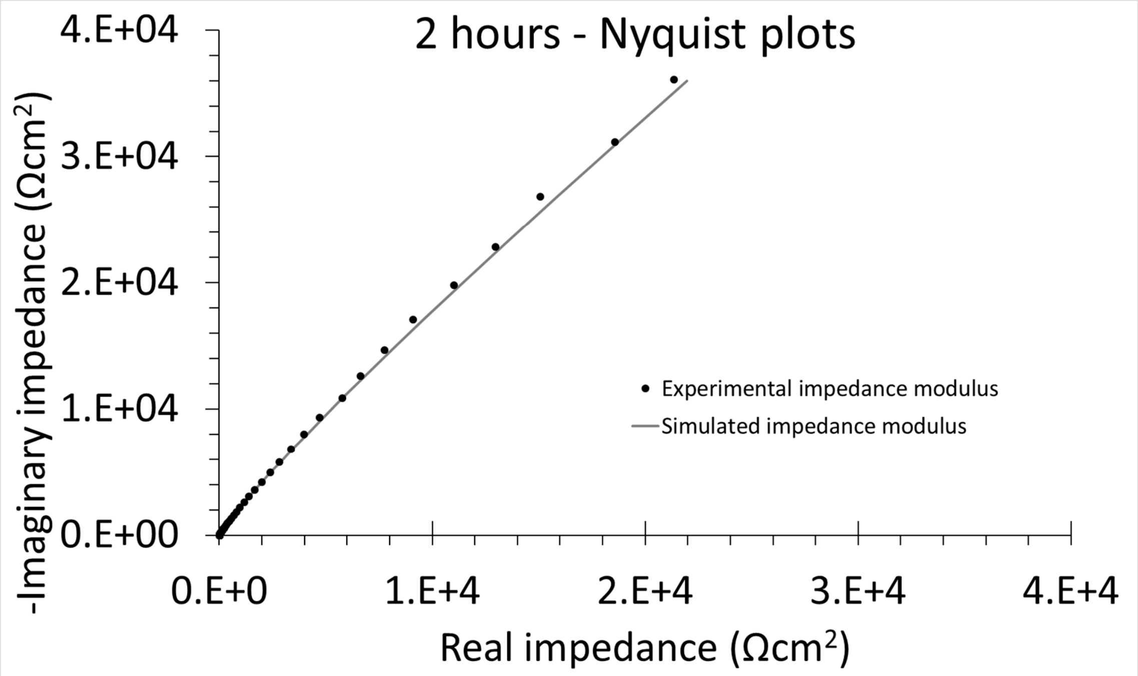
2 hours - Nyquist plots
-Imaginary impedance (Ωcm2)
Real impedance (Ωcm2)
4.E+04
3.E+04
2.E+04
1.E+04
0.E+00
0.E+0
1.E+4
2.E+4
3.E+4
4.E+4
Experimental impedance modulus
Simulated impedance modulus

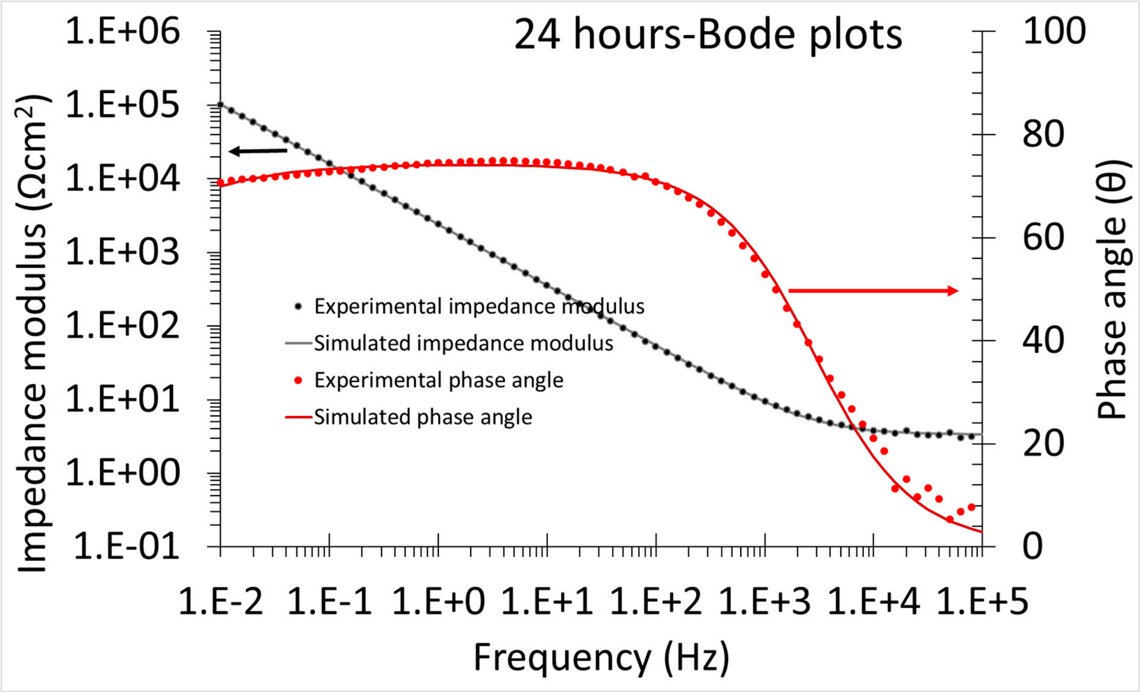
24 hours-Bode plots
Impedance modulus (Ωcm2)
Phase angle (θ)
Frequency (Hz)
1.E+06
1.E+05
1.E+04
1.E+03
1.E+02
1.E+01
1.E+00
1.E-01
100
80
60
40
20
0
1.E-2 1.E-1 1.E+0 1.E+1 1.E+2 1.E+3 1.E+4 1.E+5
Experimental impedance modulus
Simulated impedance modulus
Experimental phase angle
Simulated phase angle

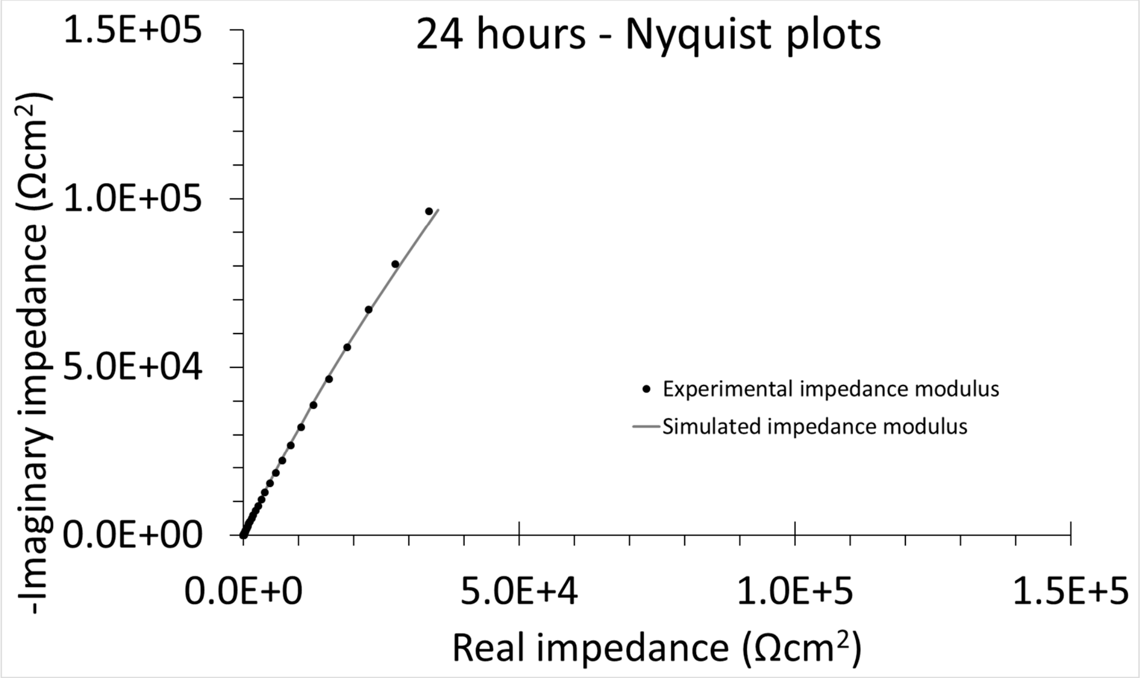
24 hours - Nyquist plots
-Imaginary impedance (Ωcm2)
Real impedance (Ωcm2)
1.5E+05
1.0E+05
5.0E+04
0.0E+00
0.0E+0 5.0E+4 1.0E+5 1.5E+5
Experimental impedance modulus
Simulated impedance modulus

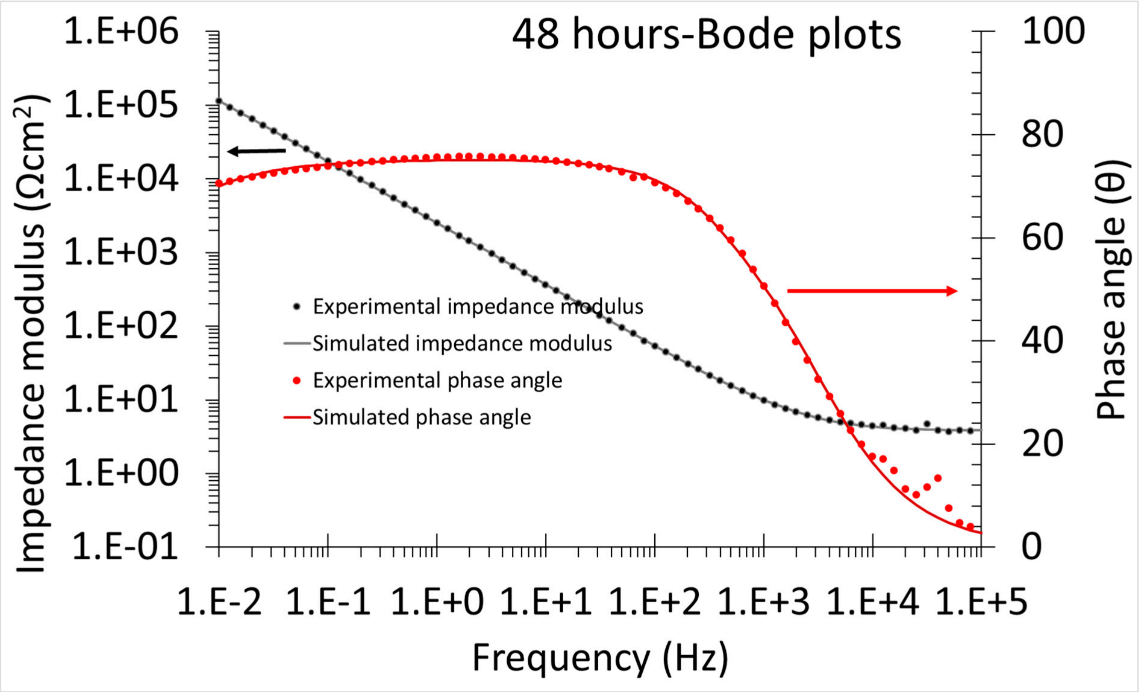
48 hours-Bode plots
Impedance modulus (Ωcm2)
Phase angle (θ)
Frequency (Hz)
1.E+06
1.E+05
1.E+04
1.E+03
1.E+02
1.E+01
1.E+00
1.E-01
100
80
60
40
20
0
1.E-2 1.E-1 1.E+0 1.E+1 1.E+2 1.E+3 1.E+4 1.E+5
Experimental impedance modulus
Simulated impedance modulus
Experimental phase angle
Simulated phase angle

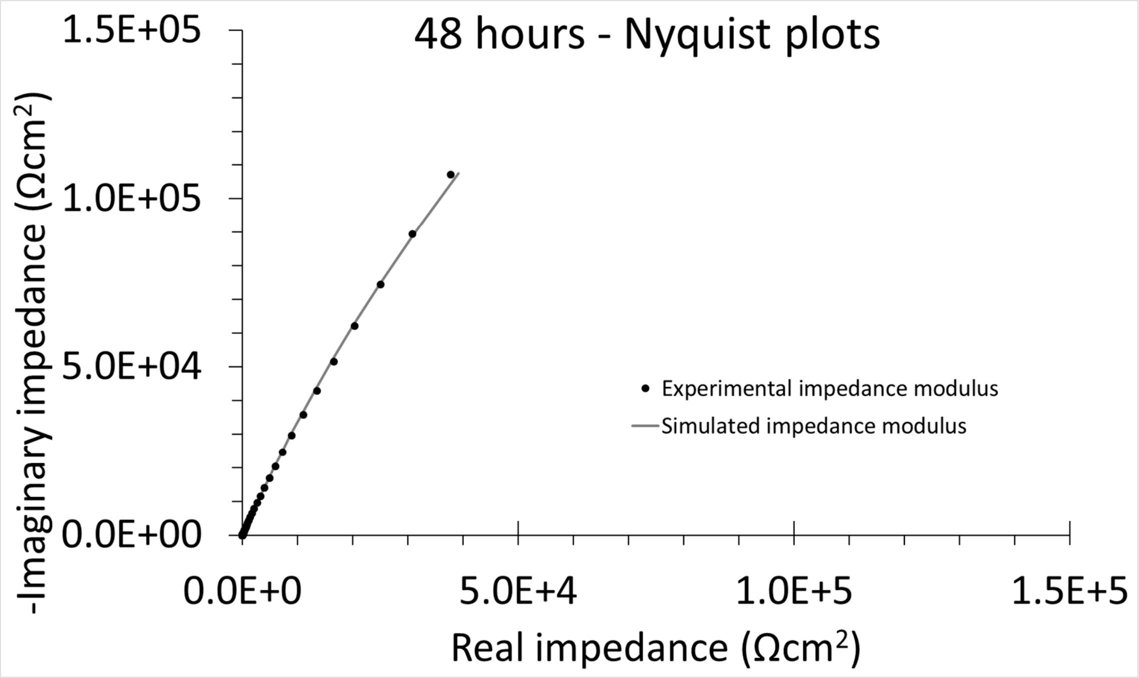
48 hours - Nyquist plots
-Imaginary impedance (Ωcm2)
Real impedance (Ωcm2)
1.5E+05
1.0E+05
5.0E+04
0.0E+00
0.0E+0 5.0E+4 1.0E+5 1.5E+5
Experimental impedance modulus
Simulated impedance modulus

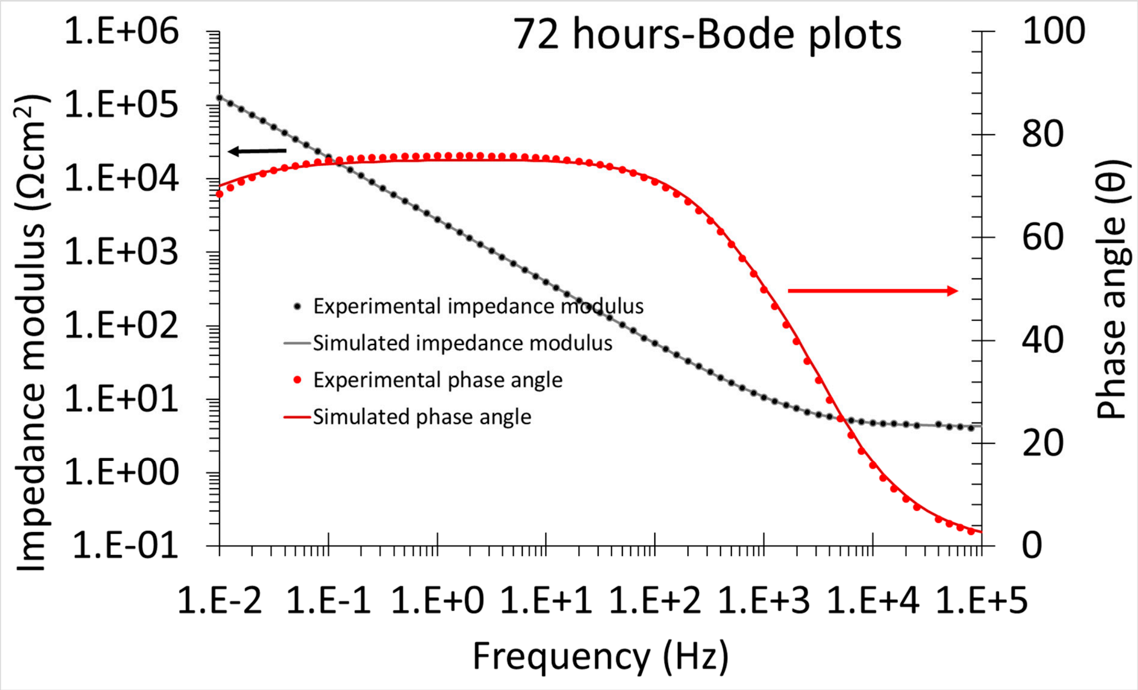

72 hours-Bode plots
Impedance modulus (Ωcm2)
Phase angle (θ)
Frequency (Hz)
1.E+06
1.E+05
1.E+04
1.E+03
1.E+02
1.E+01
1.E+00
1.E-01
100
80
60
40
20
0
1.E-2 1.E-1 1.E+0 1.E+1 1.E+2 1.E+3 1.E+4 1.E+5
Experimental impedance modulus
Simulated impedance modulus
Experimental phase angle
Simulated phase angle

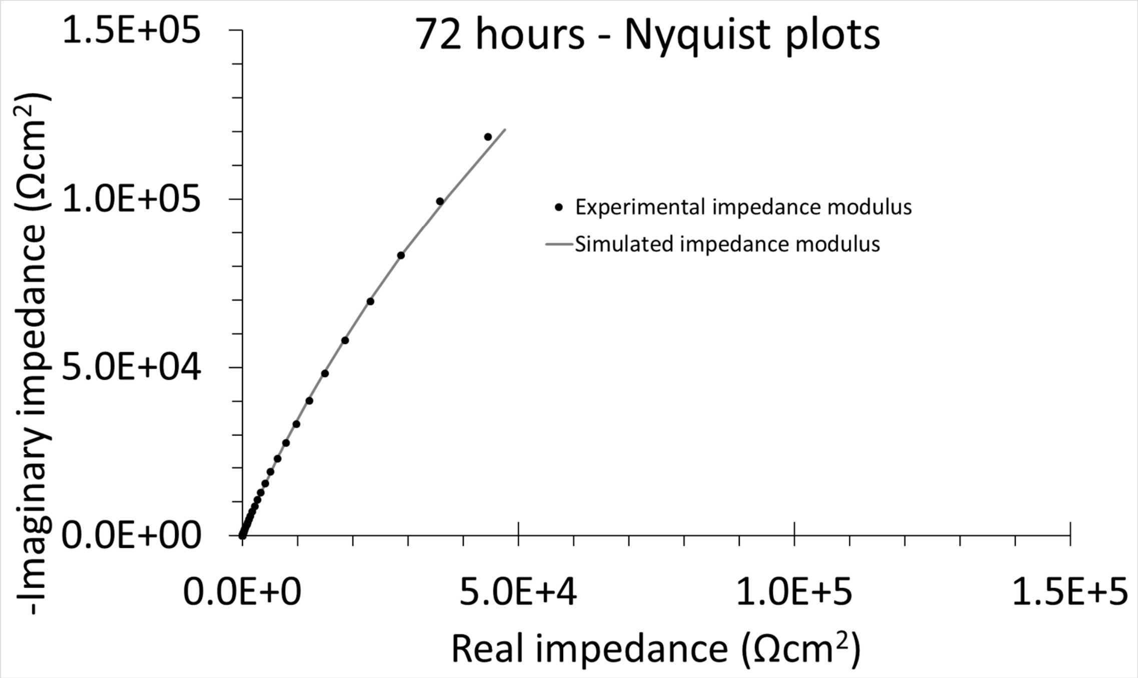

72 hours - Nyquist plots
-Imaginary impedance (Ωcm2)
Real impedance (Ωcm2)
1.5E+05
1.0E+05
5.0E+04
0.0E+00
0.0E+0 5.0E+4 1.0E+5 1.5E+5
Experimental impedance modulus
Simulated impedance modulus

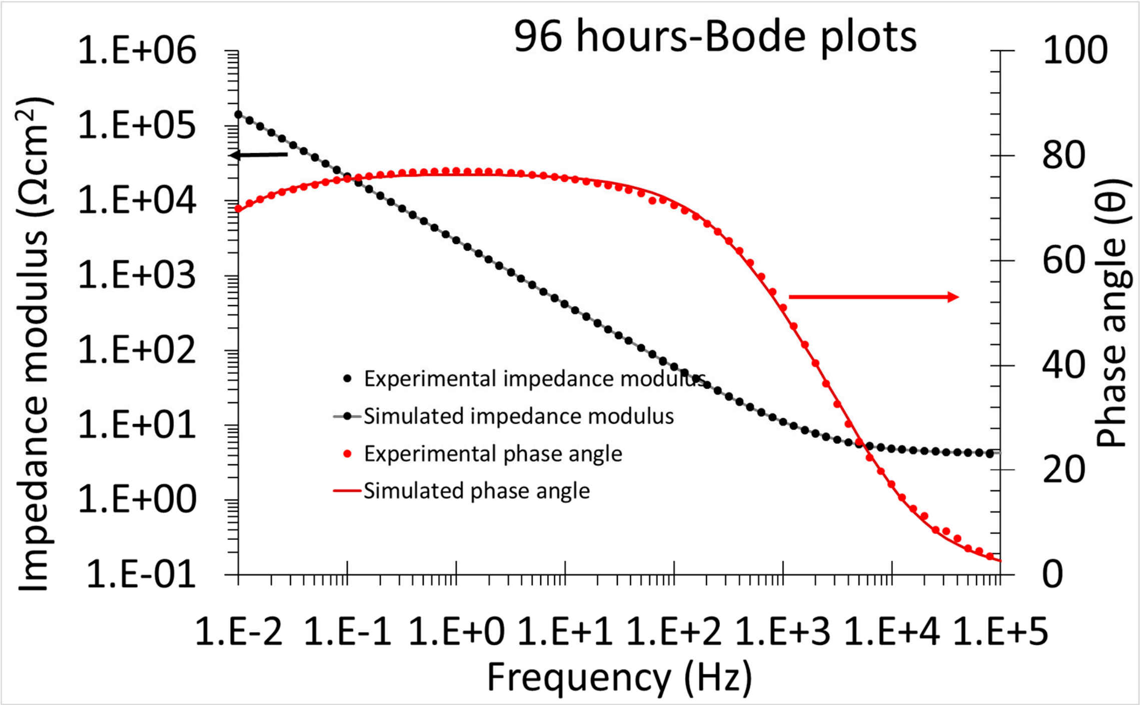
96 hours-Bode plots
Impedance modulus (Ωcm2)
Phase angle (θ)
Frequency (Hz)
1.E+06
1.E+05
1.E+04
1.E+03
1.E+02
1.E+01
1.E+00
1.E-01
100
80
60
40
20
0
1.E-2
1.E-1
1.E+0
1.E+1
1.E+2
1.E+3
1.E+4
1.E+5
Experimental impedance modulus
Simulated impedance modulus
Experimental phase angle
Simulated phase angle

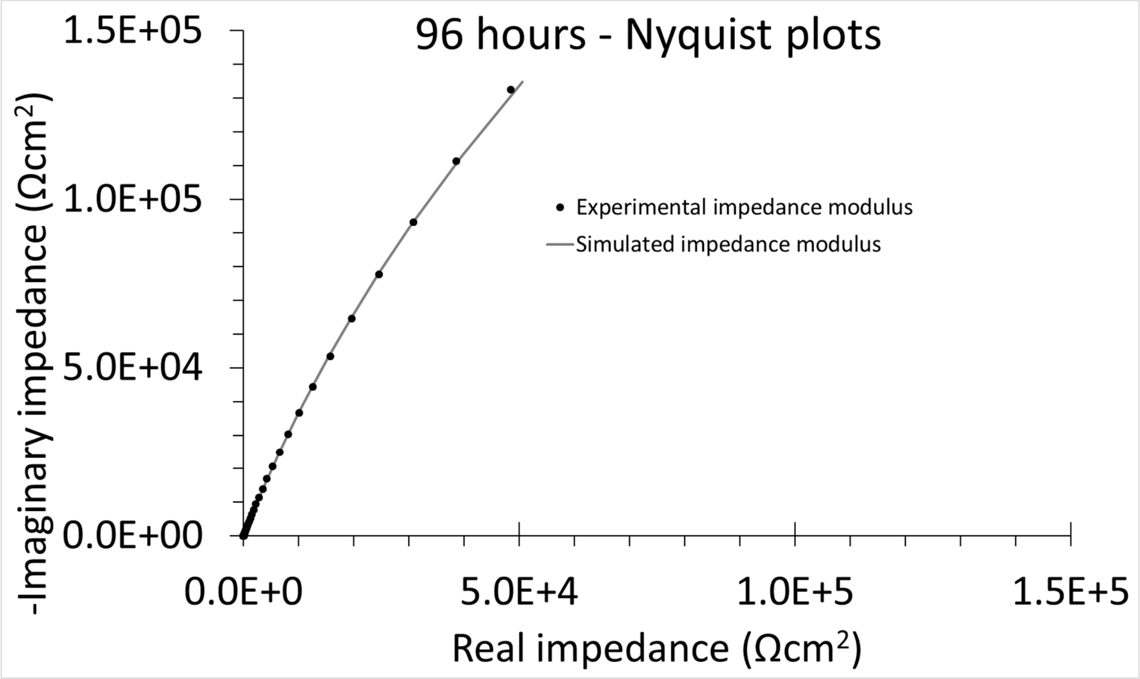
96 hours - Nyquist plots
-Imaginary impedance (Ωcm2)
Real impedance (Ωcm2)
1.5E+05
1.0E+05
5.0E+04
0.0E+00
0.0E+0
5.0E+4
1.0E+5
1.5E+5
Experimental impedance modulus
Simulated impedance modulus
